# Hidden functional complexity in the flora of an early land ecosystem

**DOI:** 10.1111/nph.19228

**Published:** 2023-08-29

**Authors:** Marco D'Ario, Brendan Lane, Marco Fioratti Junod, Andrew Leslie, Gabriella Mosca, Richard S. Smith

**Affiliations:** ^1^ John Innes Centre Norwich NR4 7UH UK; ^2^ Stanford University Palo Alto CA 94305 USA; ^3^ Technical University of Munich 80333 Munich Germany; ^4^ Center for Plant Molecular Biology‐ZMBP University of Tübingen 72076 Tübingen Germany

**Keywords:** 3D reconstructions, early land plants, finite element method (FEM), first land ecosystems, functional paleobotany, mechanical stress, plant evolution

## Abstract

- The first land ecosystems were composed of organisms considered simple in nature, yet the morphological diversity of their flora was extraordinary. The biological significance of this diversity remains a mystery largely due to the absence of feasible study approaches.
- To study the functional biology of Early Devonian flora, we have reconstructed extinct plants from fossilised remains *in silico*. We explored the morphological diversity of sporangia in relation to their mechanical properties using finite element method. Our approach highlights the impact of sporangia morphology on spore dispersal and adaptation.
- We discovered previously unidentified innovations among early land plants, discussing how different species might have opted for different spore dispersal strategies. We present examples of convergent evolution for turgor pressure resistance, achieved by homogenisation of stress in spherical sporangia and by torquing force in *Tortilicaulis‐like* specimens. In addition, we show a potential mechanism for stress‐assisted sporangium rupture. Our study reveals the deceptive complexity of this seemingly simple group of organisms.
- We leveraged the quantitative nature of our approach and constructed a fitness landscape to understand the different ecological niches present in the Early Devonian Welsh Borderland flora. By connecting morphology to functional biology, these findings facilitate a deeper understanding of the diversity of early land plants and their place within their ecosystem.

## Abstract 2

See also the Commentary on this article by Niklas, **241**: 523–524.

## Introduction

The colonisation of land by plants is one of the most important events in the history of our planet. These plants and their descendants shaped all subsequent land ecosystems, forming the basis for terrestrial primary productivity (Kenrick *et al*., 2012) and likely altering global sedimentation patterns, biogeochemical cycling and perhaps oxygenation levels (Algeo & Scheckler, 1998; Gibling & Davies, 2012; Morris *et al*., 2015; Lenton *et al*., 2016; Wallace *et al*., 2017; McMahon & Davies, 2018; Davies *et al*., 2021). The first land ecosystems were one of a kind – composed of diminutive plants whose reproductive structure, a sporangium subtended by a vertical axis, was rarely taller than a centimetre (Edwards, 1996). Despite such simple morphology, these ecosystems showed an outstanding diversity, with new species still being described (Edwards, 2014; Gess & Prestianni, 2021; Edwards *et al*., 2022a). Investigating the biology, ecology and physiology of early land plants is therefore crucial not only for understanding their evolution, but also for teasing apart how plants may have shaped the broader Earth system.

The basic biology of early land plants is widely thought to be similar to extant bryophytes, with a dominant vegetative gametophyte and a simple, nutritionally dependent sporophyte (Boyce, 2008; Tomescu *et al*., 2014; Edwards *et al*., 2022c). Recent phylogenetic studies of extant taxa, however, suggest that the ancestral sporophyte was more complex (Puttick *et al*., 2018; de Sousa *et al*., 2019; Harris *et al*., 2020; Clark *et al*., 2022) and that living bryophytes may not represent a good model for the first land plants (McDaniel, 2021). Some important early groups such as cooksonioids and eophytes are indeed unique relative to living taxa, combining aspects of bryophytes (e.g. extremely small sporophytes) with features of extant vascular plants such as multiple sporangia and vascular tissue (Boyce, 2008; Edwards *et al*., 2022b). Exploring the biology and ecology of these unusual groups is therefore critical to understanding the nature of early plant life.

Although diverse plant body fossils are known from the Late Silurian through Early Devonian (Li & Edwards, 1992; Edwards & Wellman, 2001; Shou‐Gang & Gensel, 2001; Kotyk *et al*., 2002; Libertín *et al*., 2018), investigating how these taxa worked as organisms can be challenging because most are found as fragmentary compressions that preserve neither the plant as a whole nor the anatomy of its parts. The few Silurian and Early Devonian terrestrial lagerstatten (Edwards, 1996; Wellman *et al*., 1998; Glasspool *et al*., 2004; Kerp, 2017) have therefore played a large role in our understanding of early plant biology and ecology. In particular, permineralisations from the Early Devonian Rhynie Chert, *c*. 407 million years ago (Ma; Edwards *et al*., 2017), have been foundational in our understanding of early plant anatomy and morphology (Kidston & Lang, 1921; Edwards, 1986; Kerp, 2017), life history strategies (Remy, 1982; Taylor *et al*., 2005), interactions with fungi (Taylor *et al*., 2003), and interactions with the substrate (Hetherington & Dolan, 2019). Slightly older deposits from the Welsh Borderland (Lochkovian; 419–410 Ma) likewise provide an important window into early terrestrial ecosystems and novel plant groups (Edwards *et al*., 2022a,b), in this case through charcoalified fossils that preserve cellular‐level anatomical detail. In this study, we take advantage of the excellent preservation found in these fossils to explore the reproductive biology and diversity of early sporophytes through biophysical modelling.

Using biomechanics to better understand the biology, ecology and physiology of fossil plants has a long history (Niklas, 1992; Bateman *et al*., 1998) ranging from topics as diverse as leaf evolution (Beerling *et al*., 2001), the physics of wind pollination (Niklas, 1983, 1985), and ancient physiology (Cichan, 1986; Wilson *et al*., 2008; Wilson & Fischer, 2011), to name a few. Here, we describe a new approach that leverages the open‐access software MorphoDynamX (www.MorphoDynamX.org) for three‐dimensional reconstruction and analysis of fossilised sporangia. Using finite element method (FEM) simulations, we highlight how the morphologies of some species might have used mechanical stress accumulation to assist sporangia opening. Additionally, our approach identified two convergent instances of turgor pressure tolerance, which we interpret as a more specialised adaptation towards propelled spore release. This study emphasises the previously underappreciated complexity of the Early Devonian Welsh Borderland flora, connecting morphological diversity and tissue mechanics with specialised reproductive biology.

## Materials and Methods

### Fossil specimens

All specimens analysed in this study derive from the north of Brown Clee hill locality in Shropshire, UK (Edwards *et al*., 1994). They were found in grey siltstones belonging to the *micrornatus*–*newportensis* sporomorph assemblage biozone (Richardson & McGregor, 1986), dating to the early Lochkovian stage of the Early Devonian (419.2 to *c*. 415 Ma). We investigated 15 specimens with a variety of affinities, including cooksonioids, characterised by discoidal sporangia (Edwards & Feehan, 1980; Habgood *et al*., 2002), *Tortilicaulis‐like*, characterised by twisting sporangial cell files (Fanning *et al*., 1992; Edwards *et al*., 1994), and other eophyte taxa, which produce valvate sporangia with permanent cryptospore dyads (Edwards *et al*., 2022b). All specimens are preserved as charcoalified sporangia and some likely represent new taxa, including a possible new species of *Partitatheca* (Edwards *et al*., 2012) and new genus from here on referred to as ‘Spherical sporangium’. A formal description of these taxa is beyond the scope of this study and should be the focus of later studies.

### Three‐dimensional reconstructions

Scanning electron microscope (SEM) images of 15 different fossils sporangia taken at different angles were used to generate three‐dimensional reconstructions. Cell outlines visible in the SEM images were traced using the Krita imaging software then imported for reconstruction in the open‐source software MorphoDynamX (www.MorphoDynamX.org). Gaussian blur was applied on traced images before inversion followed by ITK automated watershed segmentation. For each fossil, multiple images were oriented in 3D to match the original view angle of the SEM images. The Surface Maker software produced in this work (available online, see the Data availability section) is a mesh‐making add‐on for the open‐source software MorphoDynamX. The Surface Maker allows the user to interactively generate a surface that fits the shape described by the multiple SEM images, with the possibility of including the fourfold symmetry observed in early land plant sporangia. This ‘Cell Tissue Maker’ uses a contour to represent the cross section, and a contour for the boundary. The geometry of the surface is optimised to be composed of close‐to‐square rectangles resulting in a high‐quality triangulation, which is required to improve the convergence of FEM simulations. This is achieved by rescaling the cross section contour proportionally to the distance of the boundary contour from the axis. Some faces are pentagons or triangles instead of close‐to‐square rectangles to facilitate generation of the geometry.

Segmented cells from the 2D SEM images were projected on the surface perpendicularly from the segmented images to match the viewpoint. This process was repeated to fill the empty spaces and margins between different images that were manually corrected. Cells were extruded inward by 8 μm – the cell depth observed in fractured fossils (Edwards *et al*., 2012). The height of the epidermal cell layer may vary across specimens, due to either taphonomy or biological differences (Edwards, 2014; Edwards *et al*., 2022b). This information is not always available due to the fossilisation process (see perfectly preserved sporangia; Edwards *et al*., 1994). As we were interested in comparing the effect of morphology and cell pattern on spore release, and as epidermal thickness might affect pressure resistance, we reasoned that a constant epidermal cell layer height across simulations would yield the most comparable results. We measured the thickness of *Partitatheca cymosa* epidermis as the length of a joined cell wall, excluding cell bulging (Edwards, 2014), as this specimen was used for software development. *Partitatheca cymosa* cellular height was comparable to that of *Partitatheca splendida* and *horrida* (Edwards, 2014). Additionally, we reasoned that maturing sporogenous tissue should act similarly to a shoot meristem, which behaves like an inflated shell (Beauzamy *et al*., 2015), removing the need to simulate multiple layers of cells.

Some sporangia were so well‐preserved that cell segmentation of one image was sufficient for reconstruction (Supporting Information Fig. S1a). However, when damaged samples were missing sections of the sporangium, we leveraged the fourfold symmetry of sporangia to reconstruct missing sections of specimens. Gaps in tissue were filled using either the opposing side of the same fossil, or the same segmented image rotated or mirrored to cover the absent area (Fig. S1b). In extreme cases of damage, we focussed on reconstructing a discrete quarter of the specimen, which we considered to best represent the whole sporangium, as a template for full organ reconstruction (Fig. S1c). Although the accuracy of reconstruction varies due to the nature of the material, the methods we implemented ensured that both the overall three‐dimensional morphology and the cell patterns were faithful to the original.

The lower opening of the sporangia has a membrane to enclose the sporangial cavity. Those elements were not used in stress calculations. An alternative approach would have been to fix some elements in place at the base of the sporangium, often called Dirichlet points or Dirichlet boundary conditions. We initially implemented Dirichlet boundary conditions, but this resulted in the generation of artefactual stress around these points, as the inflating tissue pulled the sporangia base out of the original plane it sat on. Cells were smoothed by averaging the position of vertices at the cell boundary three times, using the position of nearby vertices at the cell boundary. To improve the convergence of FEM simulations, meshes were triangulated once, then triangles with an aspect ratio above two were merged with nearby triangles belonging to the same cell, and the resulting faces were retriangulated. This process was repeated three times and on average reduced the number of high‐aspect‐ratio triangles by 90%.

### Finite element method and stress quantification

**Fig. 1:**
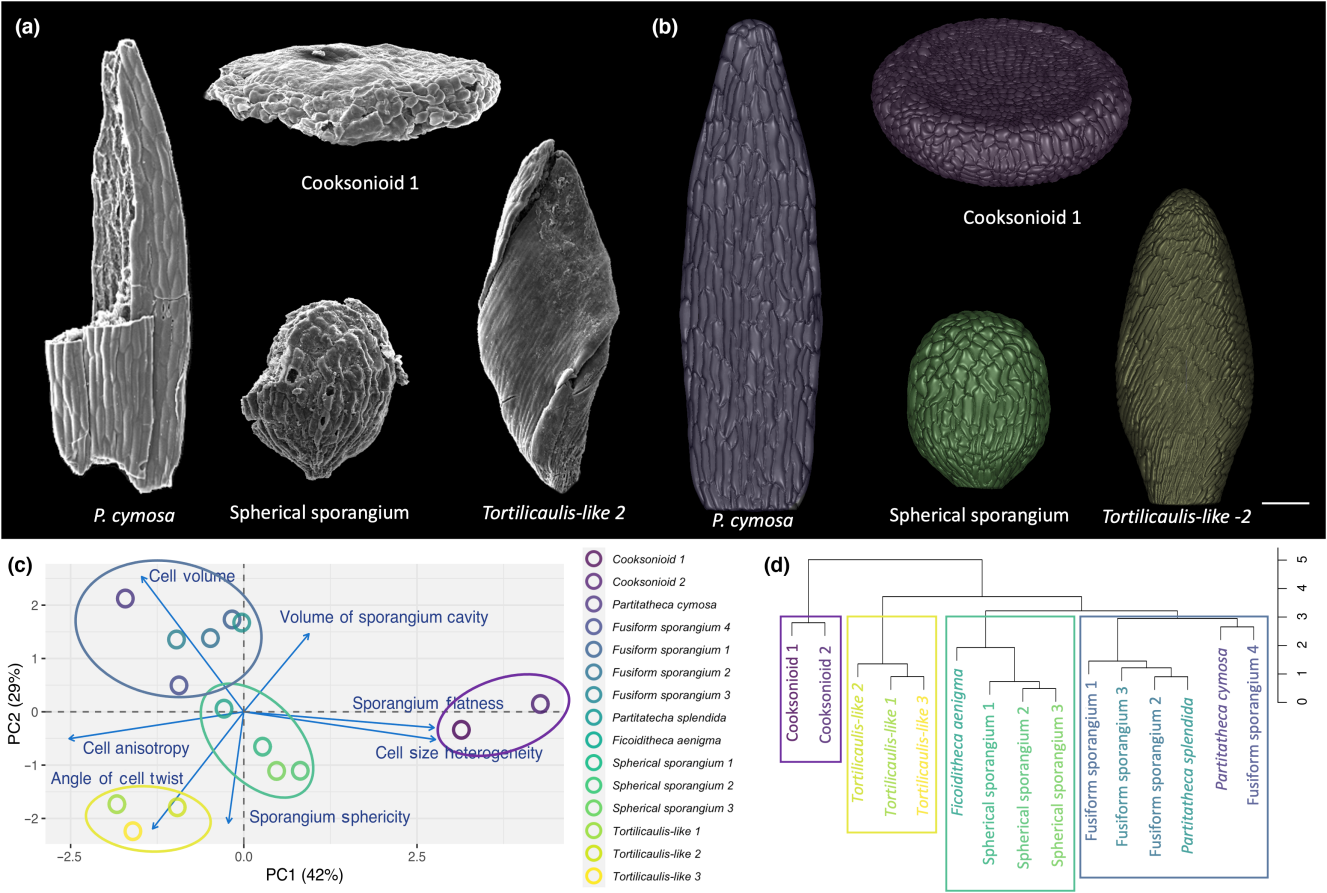
Quantitative analysis of early land plant morphology. (a) Scanning electron micrographs show different morphologies and cellular patterns of early land plants. *Partitatheca cymosa* was previously described (Edwards *et al*., 2012). (b) Three‐dimensional reconstructions of the specimens shown in (a). Reconstructions are coloured following the legend in (c). Bar, 100 μm. (c) Principal component analysis (PCA) shows clustering of different morphologies (ellipsis). The four clusters are: cooksonioids (purple, right), fusiform (blue, top), *Tortilicaulis‐like* (yellow, bottom) and spherical (green, middle). The first principal component (PC1) and the second principal component (PC2) captured 42% and 29% of the morphological diversity, respectively. (d) Morphology based dendrogram shows the four main clusters resulting from unweighted pair group method with arithmetic mean hierarchical clustering (*h* = 3). The position of the spherical sporangia suggests Eophyte affinity.

To finalise the reconstructions and counteract the effect of cell deflation during fossilisation, we pressurised cells from the triangulated meshes in a mechanical model using FEM (Sapala *et al*., 2018). Sporangial wall tissue was reinflated to reproduce cell bulging (Figs 1b, S1b) using MorphoMechanX (www.MorphoMechanX.org), an FEM add‐on for MorphoDynamX. The material properties were set to Young's modulus of 100 MPa and a Poisson ratio of 0.3, previously used as an estimate for the plant cell wall (Weber *et al*., 2015; Mosca *et al*., 2017; Long *et al*., 2020). Cell pressure was set to 0.5 MPa (Mosca *et al*., 2017). Of course, material properties vary amongst different tissues within the same plant and across species (Shah, 2013), but with the intent of comparing the effect of sporangium morphology and cellular patterns across specimens, we equalised and approximated material properties and opted for values used in previous studies of plant cells (Hervieux *et al*., 2016; Mosca *et al*., 2017; Sapala *et al*., 2018). Simulations were run using a semi‐implicit solver (Sapala *et al*., 2018) to a convergence threshold of 10^−3^ on the force norm. We then pressurised the sporangium cavity using the same basic parameters, with the pressure increased incrementally by 0.01 MPa, starting from 0 MPa and ending at 1 MPa. Finally, we also deflated the sporangial wall to approximate desiccation as the sporangium matured and dried before spore release. For this process, the sporangium cavity was pressurised to 0.2 MPa, and cells were deflated to a pressure of 0.001 MPa.

To study the fracturing process, the sporangia can be modelled as either ductile or brittle materials. Extensometers tests on hydrated cell walls show that plant tissue can be deformed plastically before failure (Zhang *et al*., 2021), indicating a ductile nature to the tissues. By contrast, less hydrated secondary, lignified cell walls have been shown to behave like brittle materials (Farquhar & Zhao, 2006). We used two different criteria to estimate the point of rupture: the von Mises criterion, appropriate for ductile materials, and the Max Stress criterion, appropriate for brittle materials. Although yield precedes fracture during the process of rupture; for simplicity, we considered those two to occur at the same moment.

The von Mises criterion states that breakage occurs when the von Mises stress $\sigma_{\tau }$, calculated as:

$$\sigma_{\tau }=\sqrt{\frac{{\left(\sigma_{1}-\sigma_{2}\right)}^{2}+{\left(\sigma_{2}-\sigma_{3}\right)}^{2}+{\left(\sigma_{1}-\sigma_{3}\right)}^{2}}{2}}$$

exceeds the stress at yielding of a uniaxial test. Here $\sigma_{1}$, $\sigma_{2}$ and $\sigma_{3}$ are the principal stress components (Bai & Bai, 2014; Burns, 2015). The Max Stress criterion states that breakage occurs when the highest of the principal stress components exceeds the same threshold. The tensile strength of plant specialised tissues varies with seed material minima reaching 8 MPa and stem secondary tissues crossing 57 MPa (Shah, 2013). However, some tissues such as leaves have a relatively consistent tensile strength *c*. 30 MPa (Shah, 2013). We measured a range of rupture pressure using tensile strength from 25 to 40 MPa and used both brittle and ductile breakage criteria. To avoid numerical artefacts, we defined rupture pressure as the smallest pressure at which the top 1% of most stressed elements met the failure criterion. As those criteria allowed us to reach similar conclusions (see the Results section), we reasoned that the elastic sporangia wall would behave similarly to the epidermis of onion or leaves, ductile materials with tensile strength of *c*. 30 MPa (Shah, 2013; Majda *et al*., 2022). This approximation allowed us to further compare the sporangia and quantify their ability to open.

To measure the effectiveness of stress to localise towards the opening site of sporangia and assist the dehiscence process, we quantified ‘stress‐assisted rupture’. This metric compares the mean stress across the entire sporangium with stress on the apex and was carried out at rupturing pressure using the von Mises criterion with tensile force equal to 30 MPa. We first selected the elements contained in the cylinder of infinite height and elliptical cross section with axes $\left|\max\left(y\right)\right|/2$ and $\left|\min\left(z\right)\right|/2$, where *y* and *z* are the position of the coordinates of the elements. Of these elements, only the superior ones (at the apex of the sporangium) were selected $\left(x>\left(\max\left(x\right)-\min\left(x\right)\right)/2\right)$ and of those, the stress distribution between inner and outer surface were compared and the highest one was chosen. This ensured that the selected elements are indeed those more likely to break at the apex of each sporangium. We subtracted the mean stress of all the elements of a sporangium from the selected apical elements. A positive value thus indicates that the highest stress elements were those at the top of the sporangium, while a negative one indicates that the highest stress was found elsewhere on the sporangium.

### Quantification of cell properties and morphology

Cell volume, its coefficient of variation (CV), cell anisotropy, and cell tilt were calculated from the three‐dimensional reconstructions after cell inflation at 0.5 MPa (Mosca *et al*., 2017) but before pressurisation of the cavity, as we expect this state to most closely resemble the *in vivo* state of the sporangia before spore release (Fig. 1a,b). For cell anisotropy and twist, the principal components of each cell's geometry (${\mathrm{PC}}_{c}$) were used. The results of this principal components analysis (PCA), which reveal the principal components of each cell, are not to be confused with the PCA we used for the morphospace (see below). ${\mathrm{PC}}_{c}$ were used to calculate as follows:

$$\text{Cell anisotropy}=\frac{2\left\|\mathrm{PC}1_{c}\right\|}{\left\|\mathrm{PC}2_{c}\right\|+\left\|\mathrm{PC}3_{c}\right\|}$$

$$\text{Cell twist}=\text{acos}|\mathrm{PC}1_{c}\cdot X|$$

X is the longitudinal axes of the sporangium, defined by the vector (1, 0, 0). The mean of the cells for each sporangium is shown in Table S1.

For quantification of the morphology, principal components of the geometry of the sporangium cavity (${\mathrm{PC}}_{l}$) were used. ${\mathrm{PC}}_{lx}$, ${\mathrm{PC}}_{ly}$ and ${\mathrm{PC}}_{lz}$ refer to the PC on the *x*, *y* and *z* axis, respectively. The results of this PCA were used to find the principal components of the sporangium cavity and are also not to be confused with the morphospace (below). The measurements were carried out as follows:

$$\text{Sporangium sphericity}=\frac{\sqrt[{3}]{\frac{3}{4\pi }\text{sporangium cavity volume}}}{\sqrt[{2}]{\frac{\text{sporangium cavity surface area}}{4\pi }}}$$

$$\text{Sporangium flatness}=\frac{\left\|{\mathrm{PC}}_{ly}\right\|+\left\|{\mathrm{PC}}_{lz}\right\|}{2\left\|{\mathrm{PC}}_{lx}\right\|}$$

### Morphospace and clustering

A morphospace was built using quantification of cellular properties and morphology. Principal components analysis was used to classify and cluster the specimens analysed, using the attributes listed on the top panel of Table S1. The calculations were performed with the function *prcomp* of the R package stats (v.4.2.1) with default parameters. The seven‐dimensional morphospace captured > 70% of the morphological diversity with two dimensions (PC1 = 42%, PC2 = 29%) (Fig. 1b). *k*‐Means and Partitioning Around Medoids (PAM) statistics were used to define five clusters and four main morphological clusters (Lloyd, 1982; Kaufman & Rousseeuw, 2009). Spherical sporangia form the highest confidence cluster with *Ficoiditheca aenigma* (PAM Si > 70%, *k*‐means Si > 60%) (Fig. S2; Morris *et al*., 2012). The second most confident cluster was composed *Tortilicaulis‐like* sporangia (Si > 50) (Edwards *et al*., 1994). The two cooksonioids clustered together with PAM (Si > 20%) statistics but not *k*‐means, in line with the known diversity of cooksonioids (Habgood *et al*., 2002). The rest of specimens form one (*k*‐means Si > 25% except for Fusiform sporangium 4 Si > 5%) or two clusters (PAM Si > 45%, Si > 5%), with *P. splendida*, Fusiform sporangium 1, 2 and 3 forming one of the PAM clusters, and *P. cymosa* and Fusiform sporangium 4, forming their own PAM cluster (Fig. S2). This information, combined with the height dendrogram (method = average), suggested four main morphological clusters: cooksonioid, *Tortilicaulis‐like*, spheriform, and fusiform. The latter is subdivided into two subclusters (*cymosa*‐like and *splendida*‐like) (Fig. S2). This clustering was supported by morphology‐based dendrogram using unweighted pair group method with arithmetic (UPGMA) mean hierarchical clustering (*h* = 3), carried out with the function *hclust* of the R package stats (Fig. 1d; Sokal, 1958). R was run in its stable release 4.2.1.

### Spore yield and fitness measurements

Relative spore yield was calculated as proportional to sporangial cavity volume, with the expectation that a larger sporangial cavity would have contained more spores.

To measure fitness, we first normalised each of the three attributes using the following equation:

$$f\left(\mathrm{at}\right)=\frac{\mathrm{at}-\min\left(\mathrm{AT}\right)}{\max\left(\mathrm{AT}\right)-\min\left(\mathrm{AT}\right)}$$

at is the value for the attribute of one specimen and AT is the collection of one of the attributes of each sporangium. In this way, the lowest attribute value is equal to 0 and the maximum value is equal to 1, and the linear relationship amongst sporangia attributes is preserved. To score fitness, we calculated the Euclidean distance of each point from the origin as follows:

$$f=\sqrt{\frac{\sum_{i}{\mathrm{at}}_{i}^{2}}{3}}$$

so, the further away a specimen is from the origin, the higher its fitness. Note, we divided by 3 so that the highest possible fitness, achieved with all attributes equalling 1, would also equal to 1.

### Figures and statistical analysis

All graphs were produced, and statistical analyses performed, in R.

## Results

### Sporangia reconstruction

Three‐dimensional charcoalified fossils are scarce, and most holotypes described have been destructively sampled with the intent of studying spore affinity and morphology (Morris *et al*., 2012; Edwards, 2014). Thus, we were eager to develop a software capable of non‐invasive quantitative fossil reconstructions not only on previously undescribed specimens, like the spherical sporangia (Fig. S3), but also with previously published 2D SEM images of damaged sporangia (Edwards *et al*., 1994; Morris *et al*., 2012; Edwards, 2014; Figs 1a, S1). Virtual surfaces were generated to match the three‐dimensional shape of sporangia, and SEM image cell outlines were projected onto these surfaces (see the Materials and Methods section). This approach combined the quantitative power of image analysis with the flexibility of computational modelling to produced accurate, complete, three‐dimensional reconstructions of fossilised sporangia (Fig. 1b). These reconstructions were able to capture both the global 3D shapes of sporangia and their cellular structure and topology.

To counteract the effect of deflation, an inevitable consequence of the fossilisation process, we pressurised cells in a mechanical model using FME (Sapala *et al*., 2018; Figs 1b, S1b; see the Materials and Methods section). Tissue was reinflated to reproduce cell bulging using previously established parameters for cell wall material properties (Mosca *et al*., 2017; Sapala *et al*., 2018). Conceivably, this facilitated a closer resemblance to how the specimens looked when they were alive (Figs 1b, S1b).

### Four distinct morphologies revealed by morphospace analysis

To study species diversity, we constructed a morphospace using quantitative analysis of their characteristics, describing sporangia morphology and cell properties (Table S1). Principal components analysis and hierarchical clustering revealed four major morphological groups (Figs 1c,d, S2). Outgroup to the rest of the specimens, the cooksonioids formed the first group – distinct for their discoidal flat morphology, cell size heterogeneity, and cell shape isotropy (Fig. 1c). The second group was composed of the previously described genus *Tortilicaulis* (Edwards *et al*., 1994), characterised by highly elongated cells which form an angle with the central axis between 30° and 40° (Fig. 1b,c; Table S1). Forming the third morphological group, fusiform sporangia and members of the genus *Partitatheca*, also included the previously described *P. splendida* and *P. cymosa* (Edwards *et al*., 2012, 2022a; Edwards, 2014). In this group, *P. cymosa* and fusiform sporangium 4 form a subcluster (Fig. 1d). Sporangia belonging to the fourth and final group were characterised by small cavities and spherical morphology and form a cluster with the previously described *F. aenigma* (Morris *et al*., 2012; Fig. 1c,d). Clustering of spherical sporangia with *F. aenigma* suggests an affinity with the eophytes. To understand the link between morphological diversity and functional biology, we investigated how shape and cellular arrangement affected the mechanical properties of our virtual sporangial tissues.

### Pressure tolerance and spore release

Beyond the role in spore development, sporangia also play a role in spore dispersal. Propelled propagule dispersal is a highly derived trait, which has evolved multiple times in the tree of life. Regardless of mechanism, the tissue must withstand mechanical stress generated by the turgor pressure necessary to build tensile forces (Schneller *et al*., 2008; Whitaker & Joan, 2010; Noblin *et al*., 2012; Hofhuis *et al*., 2016). In *Cardamine hirsuta*, the pressurised cells along the seedpod force valve coiling (Hofhuis *et al*., 2016); in *Sphagnum*, collapse of the sporangial wall causes a rise in internal pressure (Whitaker & Joan, 2010); and in the catapulting mechanism of ferns, sporangia‐negative pressure is generated by water evaporation that forces bending of the whole annulus (Noblin *et al*., 2012). In our system, much less complex than these examples, the simplest hypothesis would be that tensile force is generated as the result of growth and released as the epidermis dries (Edwards *et al*., 2022b). In plants, growth is also a turgor pressure‐driven process, where organs act like inflated shells, effectively behaving like inflated sporangia, at pressure reaching up to 1 MPa (Beauzamy *et al*., 2015). Therefore, we studied mechanical stress accumulation as the result of changes in internal pressure of the sporangial cavity, with the assumption of an ability to withstand higher pressure and generating higher tensile forces as they grow, and thus releasing spores at further distances.

**Fig. 2:**
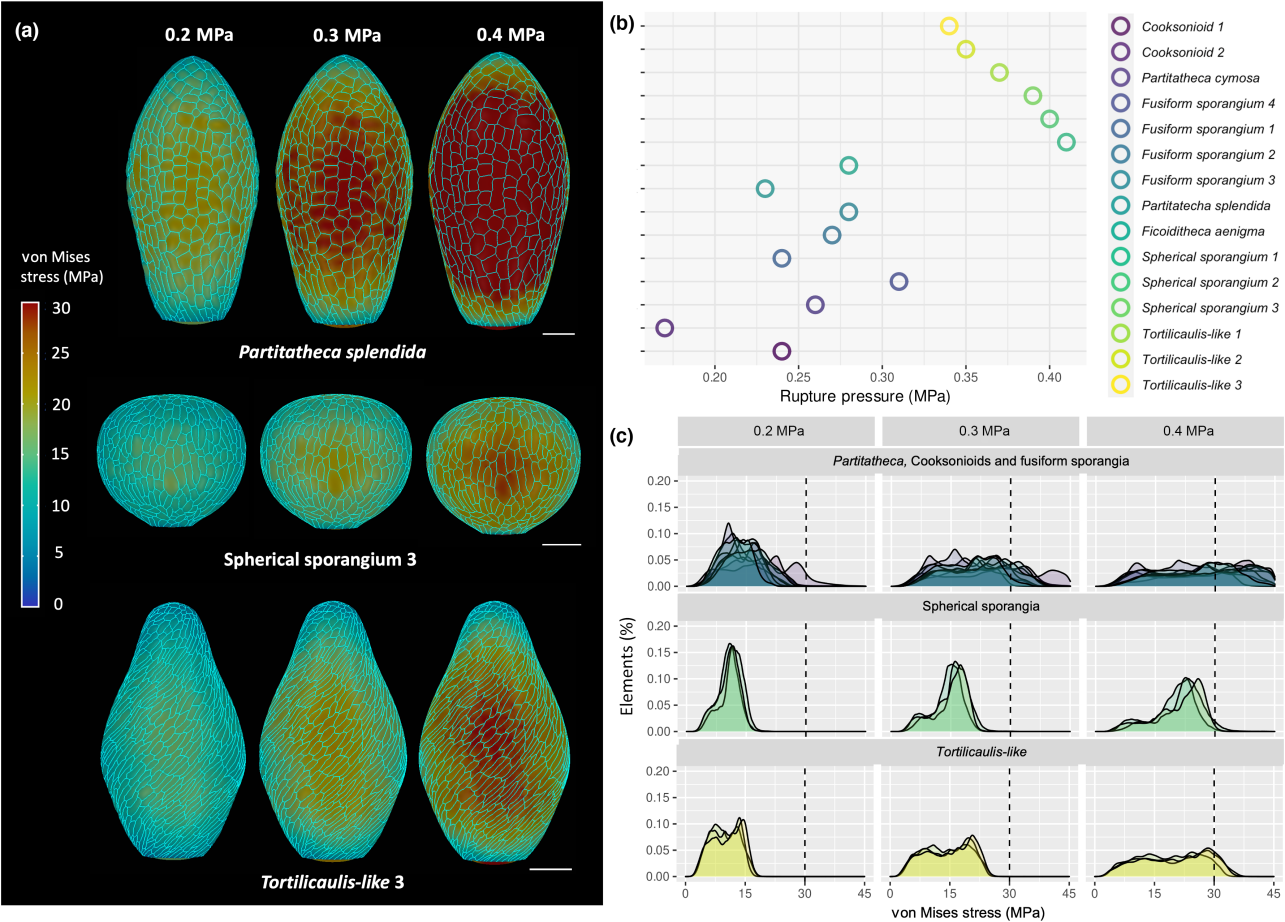
Pressure tolerance in early land plants. (a) Finite element method simulations show stress accumulation at different internal pressures (0.2, 0.3 and 0.4 MPa). Bars, 100 μm. (b) Pressure of rupture for each specimen using von Mises criterion and a tensile strength of 30 MPa (Shah, 2013; Zhang *et al*., 2021). Spherical sporangia could withstand internal pressure of *c*. 0.4 MPa whilst *Tortilicaulis‐like sporangia* averaged just > 0.35 MPa. Those pressures are well above the known pressure required for spore propulsion (Whitaker & Joan, 2010). The rest of the specimens analysed barely cleared 0.3 MPa. (c) Stress distributions of the tissue of each specimen at different internal pressures (0.2, 0.3 and 0.4 MPa). The dashed line represents the breakage point following the von Mises criterion (30 MPa). The increase in distribution spread in fusiform, *Tortilicaulis‐like*, and cooksonioid sporangia is what causes rupture. This contrasts with spherical sporangia where stress is more homogeneous across the tissue. Legend for (b, c) is shown at the top right.

We studied how stress accumulated at subcellular level by simulating different pressures of the sporangial cavity ranging from 0 to 1 MPa, to cover the equivalent known range of pressure in plants that use propelled spore ejection (Schneller *et al*., 2008; Whitaker & Joan, 2010; Noblin *et al*., 2012; Hofhuis *et al*., 2016; Fig. 2a; Video S1). We utilised both the von Mises failure criterion for ductile material and the Max Stress criterion for brittle material (Bai & Bai, 2014) to estimate rupture pressure as the highest pressure that a specimen could withstand (Figs 2b, S4). The criteria state that failure occurs when stress exceeds the uniaxial test stress at yielding. For both criteria, spherical and *Tortilicaulis‐like* sporangia performed best (Fig. S4). We also explored a set of tensile strengths for each criterion, from 20 to 40 MPa (Fig. S4). Likewise, spherical and *Tortilicaulis‐like* sporangia performed the best across the whole range, scoring best at maximum strength (40 MPa) and minimum strength (20 MPa). Noteworthy, fusiform sporangium 4 had comparable performance at high tensile strengths but did not perform as well at low strengths (Fig. S4). Thus, using different threshold or different criteria resulted to similar comparison among the sporangia tested. To carry on with our analysis, we choose the von Mises criterion and a tensile strength of 30 MPa, with the assumption that sporangia epidermal cells behave similarly to leaves or onion skins (Shah, 2013; Majda *et al*., 2022; Fig. 2b; see the Materials and Methods section). With these assumptions, the specimens analysed were able to withstand pressure between 0.2 and 0.4 MPa, matching the range required for propelled spore ejection (Fig. 2b; Nawaschin, 1897; Yafetto *et al*., 2008; Whitaker & Joan, 2010). This supports the idea that diversity in sporangia morphology can impact the tensile force generated within the tissue, affecting the force of spore release.

Although our approach gives insight into how well each morphology can generating tensile forces, it cannot unravel the mechanism those plants might have employed for spore propulsion. Our aim was not to describe such mechanism(s), but instead to test the feasibility of the well‐accepted hypothesis of a drying‐driven mechanism of sporangial opening (Edwards *et al*., 2022b). We simulated drying of the epidermal cell layer by reducing the cell turgor from the initial 0.5 to 0.001 MPa (0 MPa was not computationally possible). Indeed, we found an increase in the highest stress, consistent with a drying‐driven opening mechanism (Fig. S5). However, the cooksonioids were the only example where the highest stress was relaxed after drying (Fig. S5). When combined with the observation that those specimens cannot withstand high internal pressure (0.24 and 0.17 MPa), this suggests that sporangia with this morphology might not propel their spores for dispersal upon drying. Another interpretation would be that the cooksonioid opening mechanism was not a drying‐driven one. Conceivably, shrinkage of the cell wall, both isotropically and anisotropically, serve as a mechanism for tensile force release and opening. Unfortunately, our simulation approach could not facilitate testing of this hypothesis. As we could not test all plausible mechanisms for tensile force release, we conclude that drying of the epidermal cell layer is consistent with a mechanism for pressure‐driven tensile force generation for all but cooksonioid morphologies (Fig. S5).

Simulations of the spherical and *Tortilicaulis‐like* sporangia showed considerable tolerance of the highest internal pressure (Fig. 2b). Stress accumulation in spherical vessels is directly proportional to the internal pressure and the size of the container (Bassel *et al*., 2014; Mosca *et al*., 2017); thus, one possibility is that the smaller sporangia cavity of *Tortilicalis‐like* and spherical sporangia is responsible for the higher pressure tolerance. However, Fusiform sporangium 4 does not show the same pressure tolerance (Figs 2, S4), despite having the smallest volume of all sporangia and being half the size of twisted and spherical sporangia. To better understand pressure tolerance in spherical and *Tortilicaulis‐like* sporangia, we studied how stress accumulated with changing pressure using the von Mises criterion and evaluating for sporangia rupture when the top 1% most stressed elements crossed the threshold (see the Materials and Methods section). Rupture can occur in two ways as pressure increases in our simulations: either the stress across the sporangium increased homogeneously in all the elements, resulting in the stress distribution to move rightwards without changes in shape; or stress changed locally and heterogeneously, with the highest stressed elements becoming more and more stressed. In this latter scenario, the distribution of stresses would appear expanding, as it widens. This latter phenomenon was observed in the stress distribution of nonspherical sporangia (cooksonioid, fusiform, and *Tortilicaulis‐like*) where the increase of stress variability across the tissue is the driver of tissue failure (Fig. 2c; Table S1). By contrast, stress accumulation in spherical sporangia occurred more evenly (Fig. 2c; Table S1) with stress distribution shifting rather than widening. To quantify this observation, we measured the change in heterogeneity of stress along the sporangia using the CV. In spherical sporangia, stress was homogenous with a CV contained between 0.27 and 0.29 during pressurisation. In nonspherical sporangia, the CV of stress ranged from 0.30 to 0.51 (Fig. 2c; Table S1), showing increased heterogeneity in stress accumulation. Therefore, spherical sporangia combat the force generated by turgor pressure by homogenising stress across the tissue instead of allowing stress to build up locally. Interestingly, a similar mechanism did not account for *Tortilicaulis* pressure tolerance, as pressure still induced higher stress variability (0.36–0.41 stress CV) (Fig. 2b; Table S1). Intrigued by this observation, we sought to investigate the nature of *Tortilicaulis* mechanism for pressure tolerance.

### Torquing force counteracts internal forces in *Tortilicaulis‐like* sporangia

**Fig. 3:**
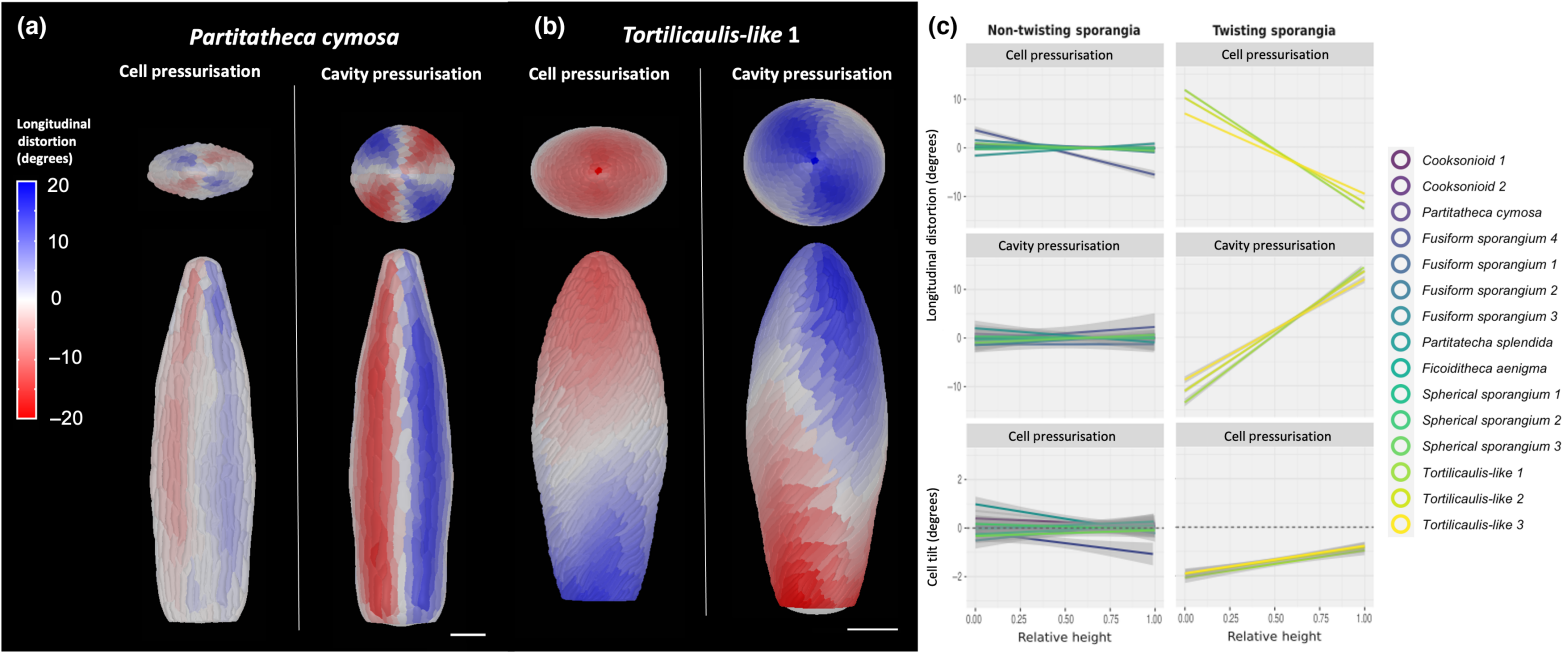
*Tortilicaulis‐like* sporangia combat stress accumulation through torquing force. (a, b) Longitudinal distortion in the non‐twisting *Partitatheca cymosa* and in the twisting *Tortilicaulis‐like* 1, respectively. Top views are shown in the top panels and front views in the bottom panels. The cell pressurisation stage is shown on the left and the sporangium cavity pressurisation stage on the right. Bars, 100 μm. (c) Linear regressions of longitudinal distortion and cell tilt over the height of the sporangium, for nontwisting (left) and twisting sporangia (right). Grey shadow represents a 99% confidence interval of the mean. During cell and cavity pressurisation, distortion in nontwisting sporangia is the same at all heights, resulting in no net rotation along the length of the sporangium. Distortion in twisting sporangia, however, accumulates over the height, resulting in anticlockwise torsion during cell pressurisation and clockwise deformation during cavity pressurisation. The bottom panel shows overall cell tilt; no tilt was observed in nontwisting sporangia, while cells in *Tortilicaulis‐like* sporangia tilted at a uniform 1–2° during the cell pressurisation stage.

Unlike the other sporangia, as cells of *Tortilicaulis‐like* sporangia were pressurised, we observed a unique torquing motion across the tissue (Video S2). *Tortilicaulis‐like* sporangia showed a phase of constriction upon cell pressurisation followed by one of expansion when the sporangial cavity inflated (Video S2). We measured distortion against the longitudinal axes to quantify this phenomenon (Fig. 3). As fossilisation captured sporangia in a deflated state, this metric evaluates the combined result of expansion away from the plane of deflation (Fig. 3a) as well as the torquing motion around the longitudinal axes (Fig. 3c). Nontwisting sporangia showed no overall displacement due to expansion, as indicated by the null slope of the linear regression across the longitudinal distortion (Fig. 3b; Table S1). By contrast, in *Tortilicaulis‐like* sporangia, the additional effect of torsion is observed as the deformation changes along the sporangium height, torquing anticlockwise during cell pressurisation, and clockwise during pressurisation of the cavity (Fig. 3c,d). The extent of visible torsion may not have been appreciable *in vivo*, as cell and sporangial cavity pressurisation would have occurred concomitantly during growth. Rather, the tissue deformation generated by cell pressurisation would have acted against the deformation generated by expansion of the sporangial cavity, reducing the resulting stress as the two forces operated against each other.

To quantify the contribution of individual cells in the twisting motion observed in *Tortilicaulis*, we computed the rotation of the longest dimension of each cell as it pressurises (Fig. 3b,d lower panels). A small change in tilt was observed for both twisting and nontwisting sporangia (Fig. 3c left); however, cells in *Tortilicaulis* rotated synchronously in the same direction (Fig. 3c right). This indicates that the observed twisting motion is the collaborative effort of each cell and highlights the role of cells in tissue mechanics. The contribution of each is minimal but the combined effort can affect the stability of the organ, manifested in this case as reduction in the stress generated by turgor pressure. Cellular coordination in *Tortilicaulis‐like* sporangia also shows an example of convergent evolution with spherical sporangia, which achieve similar pressure tolerance via different mechanisms.

### Mechanical stress‐assisted rupture

**Fig. 4:**
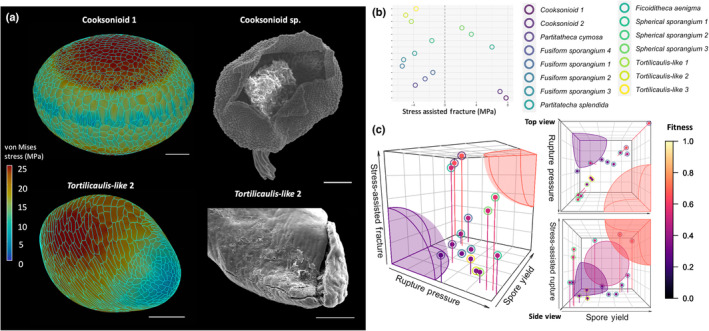
Stress‐assisted fracture and fitness landscape of the Early Devonian Welsh Borderland. (a) Comparison between fracturing pattern of fossilised specimens (right) and three‐dimensional reconstructions (left). In cooksonioids, von Mises stress accumulation coincides with the observed fracturing site (top panels), suggesting a mechanism for stress‐assisted fracturing. By contrast, in other specimens, like shown for *Tortilicaulis*, the site of rupture is complementary to the highest stress. Bars, 100 μm. (b) Stress‐assisted fracture of each specimen, measured as the difference between apical and total stress along the sporangium. Cooksonioids are the most suited for stress‐assisted fracturing, with almost 8 MPa difference in stress between the apex and the rest of the sporangium. *Ficoiditheca aenigma* follows with 6 MPa. (c) Three‐dimensional fitness landscape of the first land ecosystem, with top and side views on the right. Fitness is calculated as the distance from the intersects (*x* = *y* = *z* = 0, bottom left). The highest possible score is 1 (*x* = *y* = *z* = 1, top right). The purple sphere represents all the points with distance < 0.3 from the lowest fitness, and the red sphere represents all the points with distance < 0.3 from the highest fitness. None of the specimens analysed were located within those regions, likely indicating the presence of trade‐offs. Additionally, none of the specimens were localised at the centre, which is indicated by the pink sphere in the middle of the side view. This is the sphere of centre *x* = *y* = *z* = 0.5 and radius = 0.2. This indicates that the fitness landscape of early land plants is characterised by specialised strategies of spore dispersal, and generalist approaches are absent.

In some derived plants, like in some Bryophytes, spore dispersal is assisted by specialised structures, for example the operculum – a lid lost at maturation to assist spore release (Robinson & Shaw, 1984). Such complex structures are not found in early land plants where dehiscence is instead observed in the form of valvate opening along the sporangium (Edwards *et al*., 2012; Fig. S3). We wondered whether some of the observed morphology might direct stress localisation towards the apical part of the sporangium, assisting opening and spore release in a similar fashion to the moss operculum. Indeed, some specimens showed a pattern of stress matching the dehiscence area observed on fossilised samples (Fig. 4a), whilst in others, stress accumulated complementary to the opening site (Fig. 4b). We quantified stress‐assisted rupture by computing the apical contribution of stress accumulation and compared it with total stress (Fig. 4b). This measured how much stress localises towards or away from the opening site to assist the fracturing process (the Materials and Methods section). Cooksonioid morphology was found to be consistent with stress‐assisted rupture, closely followed by *F. aenigma* (Fig. 4b). Conceivably, the flattened shape of cooksonioids and the fig‐like shape of *F. aenigma* are the reason behind apical stress accumulation. Interestingly, two spherical sporangia showed a positive score for stress‐assisted rupture, suggesting that although their morphology facilitates stress homogenisation, the stress imbalance is still directed to the apex of the sporangia (Fig. 4b). Thus, morphology alone can assist accumulation of stress at the fracture site in sporangia.

### Ecological implications of morphological diversity

Our analysis highlighted examples of convergent evolution and specialised mechanism related to spore dispersal. We sought to connect this functional diversity to different spore dispersal strategies. To do so, we constructed a three‐dimensional fitness landscape using the following critical attributes for spore dispersal (Fig. 4c): (1) spore yield, measured as a function of the sporangial cavity volume; (2) rupture pressure, a proxy for spore release distance with the assumption that high tensile force would eject spores further away; and (3) stress‐assisted fracturing, which we suggest to be proportional to the per cent of spore released (i.e. the easier to open, the more the propagule is dispersed). In this three‐dimensional landscape, the further away from the origin, the higher the fitness, with the highest fitness located at the top right‐hand corner (Fig. 4c). The main diagonal of the space (*z* = *y* = *x*) represents generalist strategies, where fitness is achieved by equal contributions from each attribute, while distance from the aforesaid diagonal corresponds to specialisation, where the contribution of some attributes is higher than that of others. Interestingly, no specimen localised to either the maximum or the minimum fitness zones (coloured spheres). This suggests a subtle complexity of the three‐dimensional fitness landscape. As most of the specimens localised away from the main diagonal and the centre (Fig. 4c, side view pink sphere), likely the flora of the first land ecosystem was characterised by a diverse array of specialised strategies. We infer that morphological diversity in early land plants was not the result of a linear gradient towards better adaptation, but rather the manifestation of a complex ecosystem wherein differently specialised dispersal strategies coexisted.

## Discussion

The first land ecosystems were unique worlds, without equal in Earth's history. The small size of the Lilliputian forests created a distinctive microenvironment and ecology (Edwards, 1996). Here, we link the diversity of those early land plants to their specialised biology in an attempt to appreciate the depth of how natural selection might have acted on this ecosystem. Eager to contrast the one‐dimensional linear path of evolution often used to depict early land plant phylogeny (McDaniel, 2021), we show that the combined action of selective pressures and mechanical constraints likely generated niche adaptations at trade‐off nexuses.

**Fig. 5:**
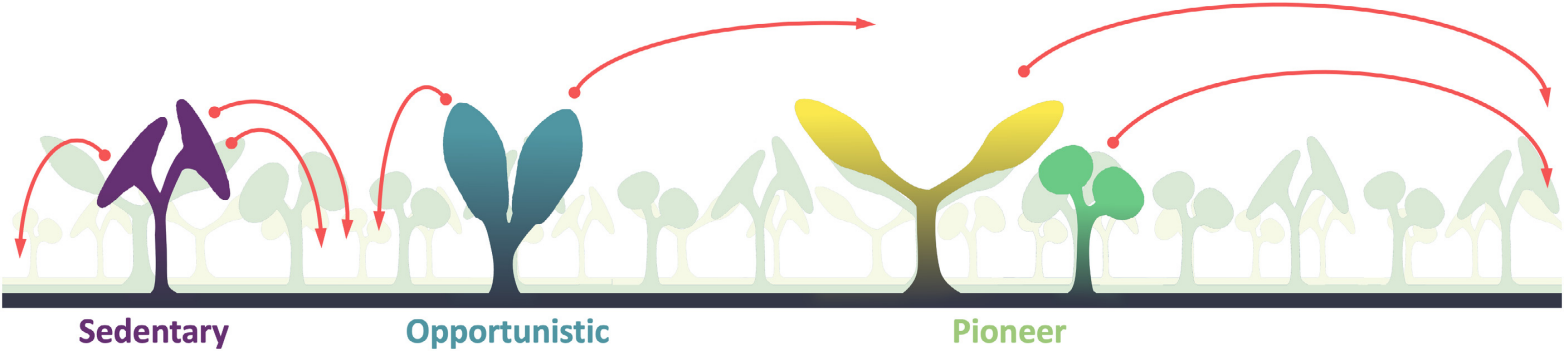
Niche allocation in early land ecosystems. Cartoon model of niche allocation in the Early Devonian Welsh Borderland, where the length of the red arrows represents the range of dispersal. The diminutive size of this ecosystem creates a unique world with three distinguishable specialised niches. Cooksonioid sporangia occupy the *sedentary* niche, adapted to produce large amount of spore for which their specialised morphology is also adapted to release with high success. At the other end of the ecosystem, the *pioneer* niche is occupied by *Tortilicaulis‐like* and spherical sporangia‐bearing plants which are convergently adapted to eject their spore further than other plants in the Early Devonian Welsh Borderland. Finally, the *Partitatheca* and other fusiform sporangia‐bearing species occupy the *opportunistic* niche by combining an above average dispersal distance and spore yield.

In the Lilliputian forests of the Welsh Borderland, marginal differences in the force of spore release would result in a considerable advantage, assisting spores to reach turbulent air (Gregory, 1973) and facilitating spores to reach available habitats with ease. Spherical and *Tortilicaulis‐like* sporangia‐bearing plants appeared to have convergently evolved a mechanism for pressure tolerance to fit this *pioneer* niche, specialised in reaching distant, uninhabited environments (Fig. 5). The expected homosporous reproductive cycle of Early Devonian spores likely gave an extra edge to these pioneers, allowing for even a single spore to reproduce via self‐fertilisation. In some fungi, cooperative spore release assists the generation of vertical airflow and greatly increases the dispersal distance (Roper *et al*., 2010). Mechanical stress plays a crucial role in this process (Roper *et al*., 2010). We found that spherical sporangia were also adapted for stress‐directed fracturing (Fig. 4b) and might have used a similar principle: releasing pressure quickly to generate a vertical airflow. It is tempting to speculate that this facilitated spherical sporangia‐bearing plants to spread their spores even further. Indeed, the size of opening would directly impact dispersal distance, with smaller opening generating higher forces with similar pressure. Complicated fluid‐dynamics simulations are required to understand the extent of the effect of this mechanism and should be the subject of future research.

Stress‐directed fracturing likely also drove apical opening of the sporangia to ensure full spore release performing an analogous function to the moss operculum (Robinson & Shaw, 1984). This seems particularly important in species adapted to produce large amounts of spores where failure in opening would squander such investments. Corroborating this idea, the cooksonioids scored best in stress‐assisted rupture and spore yield (Fig. 4b; Table S1). Those specimens also scored poorly in dispersal ability, strongly supporting a scenario in which cooksonioid‐like sporangia‐bearing plants were adapted to release a large number of spores within close proximity to the mother population (a behaviour that we refer to as *sedentary*) (Fig. 5). Although we implied that the distance of release and spore amount contributed positively to fitness, it is important to emphasise that neither strategy is always beneficial as environment plays a crucial role in determining fitness (Lande *et al*., 2009; Tarnita *et al*., 2015; Weiss‐Lehman & Shaw, 2022). When considering the minute size of those early communities, and the microenvironments within (Edwards, 1996), an intricate picture emerges in which this ecosystem was a complex network between its components and its available niche. Fusiform sporangia bearing plants and individuals of the *Partitatheca* genus showed adaptation for spore yield and dispersal whilst unable to match the potential of other genera in either trait. Such mid‐range specialisation would allow those plants to fill the available space not taken by the other plants in an *opportunistic* fashion, reaching the edge of the Lilliputian forest yet unable to reach far‐away lands (Fig. 5). The obvious unoccupied *generalist* niche was also notable (Fig. 4c), although the *F. aenigma* is the specimen that most closely would have filled this role. It will be interesting in future to understand whether this niche is in fact vacant in the Early Devonian Welsh Borderlands, or whether some undiscovered species occupied it.

Understanding the complexity of extinct ecosystems is a challenge that requires sophisticated tools. Here, we developed one such tool, used it to bring the fossil record to life and explored the functional diversity of the Early Devonian Welsh Borderland flora. This is the first report for a quantitative approach of this nature in the investigations of extinct flora. This study highlights the benefits of adopting novel quantitative perspectives and approaches in paleobotany to determine the functional biology of extinct plants in relation to their morphology, at the both cellular and organ scale.

## Competing interests

None to declare.

## Author contributions

MD designed the project, developed the reconstruction software, carried out the analysis and wrote the paper, with help from all the authors. RSS supervised the development of the software and of the project. BL provided daily support for the development of the software and analysis programs. MFJ helped with data analysis, in the production of figures, as well as providing insights into the ecological significance of the results. GM developed the FEM framework, helped with the physics of the model, and provided insights into the biological significance of the results. AL helped with referencing and ensuring that correct paleobotanical terminologies were implemented. All authors contributed intellectually to this project and were essential to the discussion and conclusion of this study.

## Supporting information


**Fig. S1** Three‐dimensional reconstructions of early land plants.
**Fig. S2** Cluster and silhouette analysis of morphological diversity of early land plants.
**Fig. S3** Eophyte and *Partitatheca* affinities of unnamed fossils.
**Fig. S4** Range and comparison of rupture criteria.
**Fig. S5** Deflation of the cell layer and stress increase.
**Table S1** Summary of statistics and measurements used in this study.

**Video S1** Sporangia pressurisation.

**Video S2** Pressurisation of *Tortilicaulis‐like 1*.
Please note: Wiley is not responsible for the content or functionality of any Supporting Information supplied by the authors. Any queries (other than missing material) should be directed to the *New Phytologist* Central Office.

## Data Availability

All scripts, meshes and raw data are available open access online at doi: 10.5281/zenodo.8231126.
