# Supplementary material for: Hidden functional complexity in the flora of an early land ecosystem
Source: New Phytol. 2023 Aug 29;241(2):937–49. doi: 10.1111/nph.19228 (PMC10952896; doi:10.1111/nph.19228)
Supplement: Supplementary file 1 — Fig. S1 Three‐dimensional reconstructions of early land plants. Fig. S2 Cluster and silhouette analysis of morphological diversity of early land plants. Fig. S3 Eophyte and Partitatheca affinities of unnamed fossils. Fig. S4 Range and comparison of rupture criteria. Fig. S5 Deflation of the cell layer and stress increase. Table S1 Summary of statistics and measurements used in this study. [file NPH-241-937-s002.pdf]

New Phytologist Supporting Information

Article title: Hidden functional complexity in the flora of an early land ecosystem

Authors: Marco D'Ario, Brendan Lane, Marco Fioratti Junod, Andrew Leslie, Gabriella Mosca & Richard S. Smith

Article acceptance date: 4 August 2023

Figure S1: Three-dimensional reconstructions of early land plants.

Figure S2: Cluster and silhouette analysis of morphological diversity of early land plants: a-b)

Figure S3: Eophyte and *Partitatheca* affinities of unnamed fossils.

Figure S4: Range and comparison of rupture criteria.

Figure S5: Deflation of the cell layer and stress increase.

Table S1: Summary of statistics and measurements used in this study.

Video S1: Sporangia pressurisation.

Video S2: Pressurisation of *Tortilicaulis-like 1*.

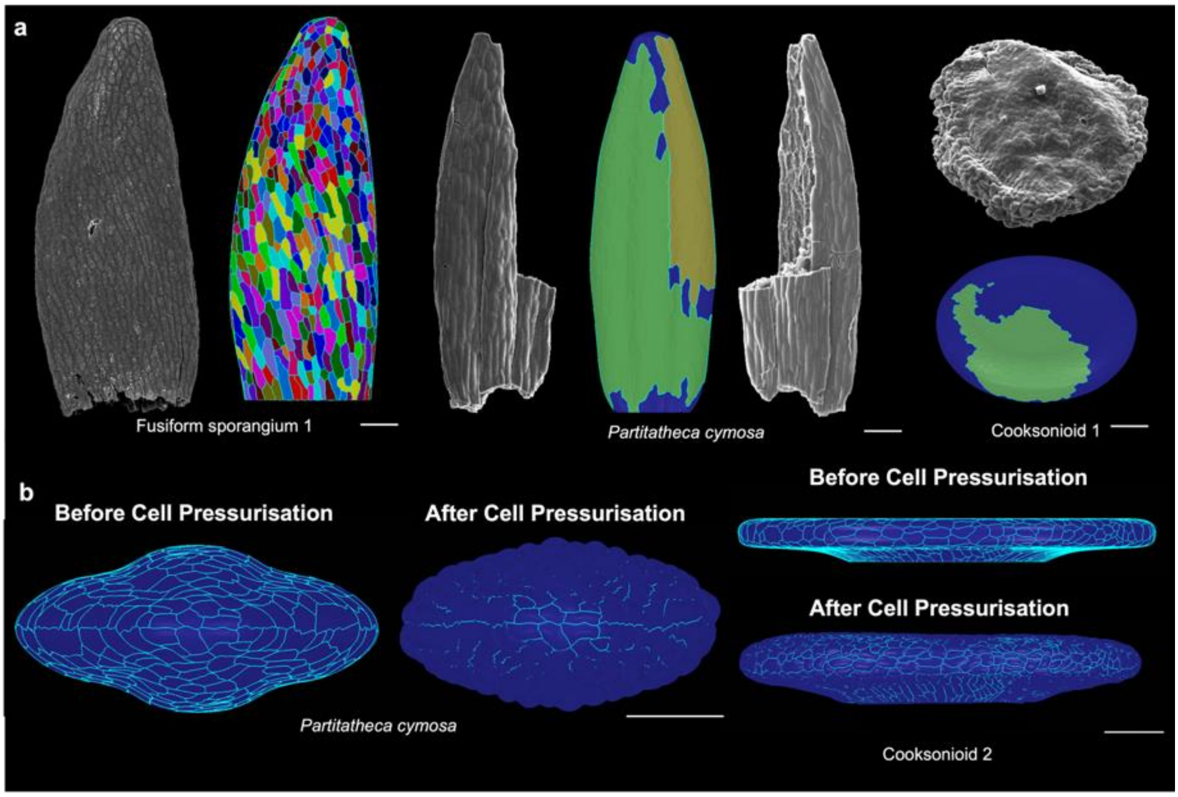

Figure S1: Three-dimensional reconstructions of early land plants. a) Comparison between reconstruction and source Scanning Electron Micrographs. In specimens like fusiform

sporangium 1, preservation of the entire sporangium allowed a direct reconstruction by one projection per side. In cases like *Partitatheca cymosa*, damaged areas were filled in using the other side of the sporangium (green vs yellow areas on the reconstruction) or by mirroring/rotating the same image. Cooksonioid 1 shows an example where only ~1/4 of the specimen was well preserved. This part is used for cell projection (green area) and morphology reconstruction, leveraging the four-fold symmetry to complete the reconstruction (only one of the SEM images used is shown). **b)** Top view of *P. cymosa* (left) and Cooksonioid 2 (right), showing the effect of cellular pressurisation. Cellular pressurisation results in reconstitution of the sporangia and bulging of the cells, more closely representing a live specimen. All scale bars = 100µm.

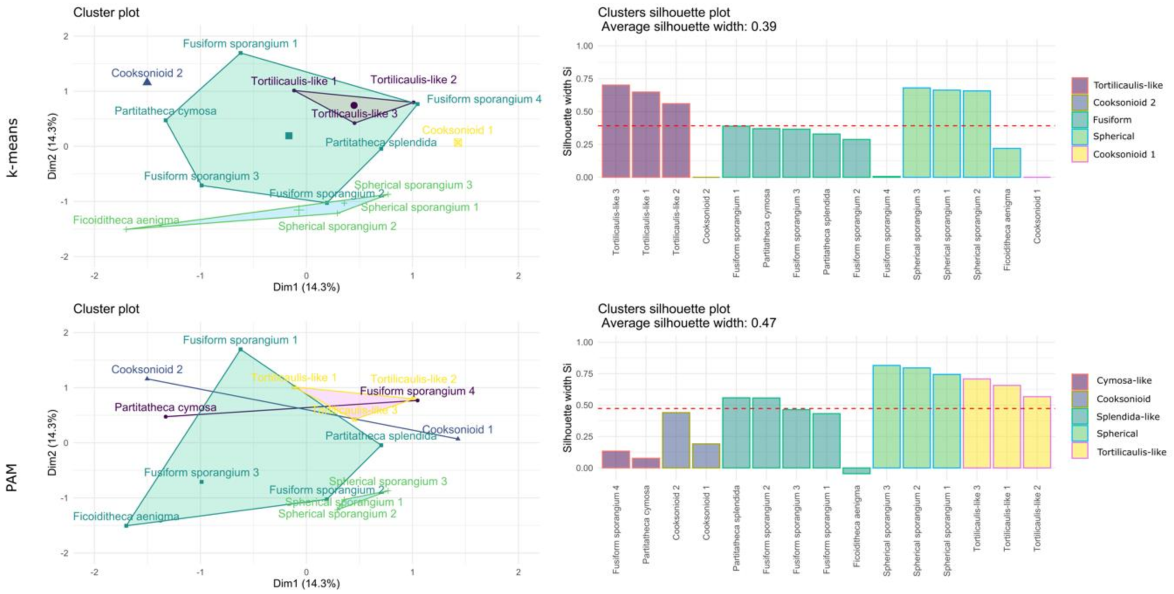

Figure S2: Cluster and silhouette analysis of morphological diversity of early land plants: a-b) K-means clustering and silhouette analysis. Using k-mean statistics, fusiform sporangia clustered together with Si between 30 and 40%, except for fusiform sporangium 4, with an Si < 5%, instead. *Tortilicaulis-like* sporangia clustered together with a high Si > 50%. Spheriform sporangia also clustered together with Si > 20% for *Ficoiditheca aenigma* and Si > 60% for the rest, and. The cooksonioids did not group together using this statistical test. **c-d)** PAM clustering and silhouette analysis. With PAM statistics, the fusiform sporangia are split into those similar to *P. splendida* or those like *P. cymosa*. The latter include *P. cymosa* and fusiform sporangium 4 with a Si > 10% and 5%, respectively. The splendida-like cluster groups fusiform sporangia (Si > 35%) and *F. aenigma*, with a negative Si. Spherical and *Tortilicaulis-like sporangia* show good grouping using PAM statistics, with Si > 75% and 55%, respectively. The two cooksonioids cluster together using PAM statistics, with Si > 45% and 20%, respectively.

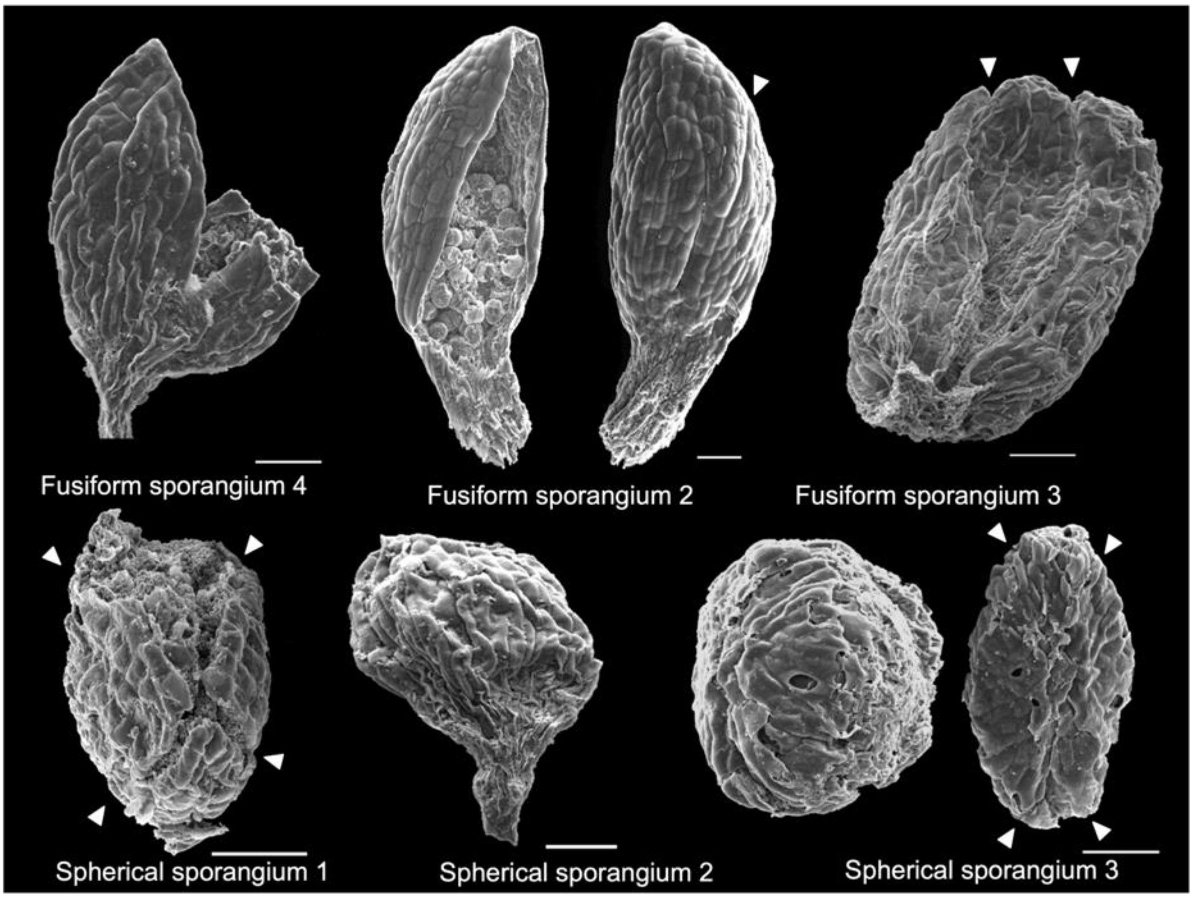

Figure S3: Eophyte and *Partitatheca* affinities of unnamed fossils. The Scanning Electron Micrographs show the location of valve aperture (arrowhead). Previously described fusiform sporangium 4 is characterised by a bivalvate opening that span along the sporangium (Edwards et al. 2022a). Fusiform sporangium 2 and 3 have visible openings (arrowhead). The tetravalvate opening in fusiform sporangium 3 is particularly evident and supports *Partitatheca* affinity. The cryptospores visible in fusiform sporangium 2 also support exophyte affinity. The tetravalvate nature of spherical sporangia 1 and 3 (arrowhead) resembles the one of *Partitatheca* and, together with the morphological clustering, would suggest affinity within the Eophytes. The arrowhead on fusiform sporangium 2 indicates a possible indehiscent line, suggesting that this specimen also was tetravalvate. Spherical sporangia *2* does not have visible openings, but its morphology is in line with the other two specimens.

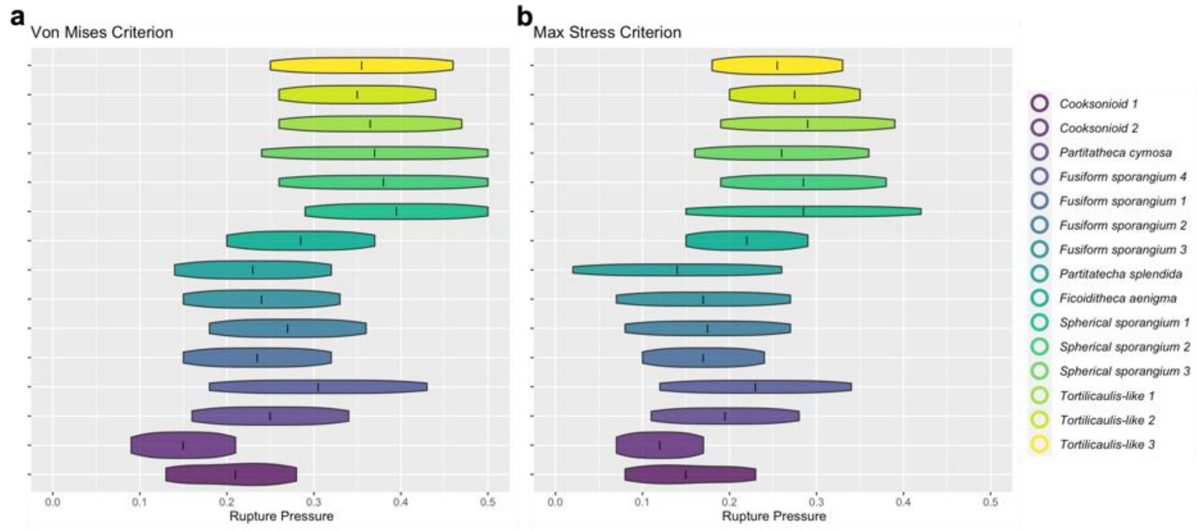

Figure S4: Range and comparison of rupture criteria. Violin plots showing the range of rupture pressure using Von Mises criterion for ductile materials (a) and Max Stress criterion for brittle materials (b). Tensile strength of the material ranged from 20MPa (rupture pressure minima) to 40MPa (rupture pressure maxima), to encompass the known ranges of tensile strength of plant materials (Shah 2013). The line in the middle of the plots represents the median across these values. Note how von Mises criterion overestimates rupture pressure, whilst the Max stress criterion underestimates it, with pressure for same tensile strength ranging around 0.1MPa. The overall trend with spherical and *Tortilicaulis-like* sporangia being the most resistant was captured by both of the criteria.

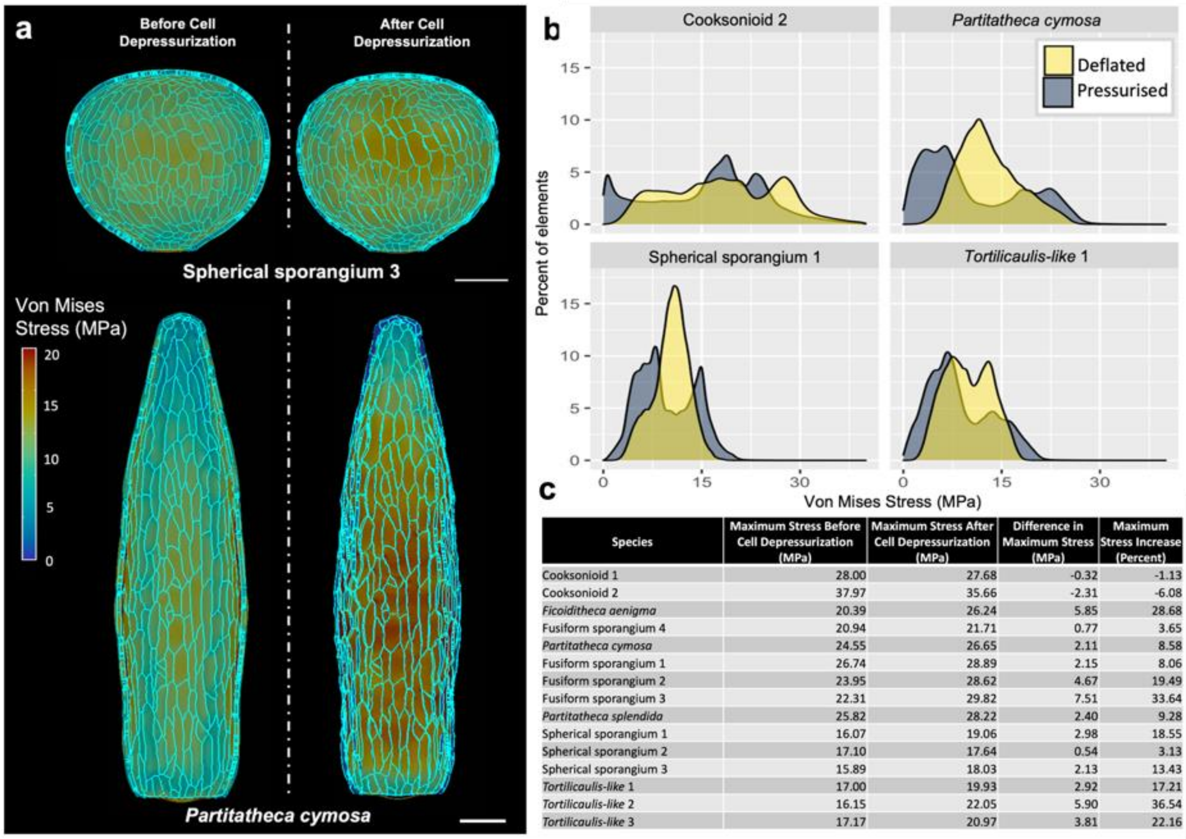

Species	Maximum Stress Before Cell Depressurization (MPa)	Maximum Stress After Cell Depressurization (MPa)	Difference in Maximum Stress (MPa)	Maximum Stress Increase (Percent)
Cooksonioid 1	28.00	27.68	-0.32	-1.13
Cooksonioid 2	37.97	35.66	-2.31	-6.08
Ficoiditheca aenigma	20.39	26.24	5.85	28.68
Fusiform sporangium 4	20.94	21.71	0.77	3.65
Partitatheca cymosa	24.55	26.65	2.11	8.58
Fusiform sporangium 1	26.74	28.89	2.15	8.06
Fusiform sporangium 2	23.95	28.62	4.67	19.49
Fusiform sporangium 3	22.31	29.82	7.51	33.64
Partitatheca splendida	25.82	28.22	2.40	9.28
Spherical sporangium 1	16.07	19.06	2.98	18.55
Spherical sporangium 2	17.10	17.64	0.54	3.13
Spherical sporangium 3	15.89	18.03	2.13	13.43
Tortilicaulis-like 1	17.00	19.93	2.92	17.21
Tortilicaulis-like 2	16.15	22.05	5.90	36.54
Tortilicaulis-like 3	17.17	20.97	3.81	22.16

Figure S5: Deflation of the cell layer and stress increase. Simulations of the drying process, showing the effect of depressurisation of the cell layer on the stress of the sporangium. **a)** Comparison between von Mises stress before and after cell depressurisation. The reconstructions are clipped so the inner wall of the sporangium cavity is exposed. The cavity was pressurised to 0.2 MPa prior to cell deflation. Scale bars = 100 µm **b)** Stress distribution showing the change in stress across sporangia of 4 different species. Note how after cell depressurisation the flank of the distribution moves to the right, indicating that the fraction of elements with the highest stress increases, consistent with a drying-driven mechanism for sporangium rupture. **c)** Table showing the change in maximum stress, calculated minimum of the 1% of elements with the highest von Mises stress. In all cases, except the cooksonioids, the highest stress increases after deflation of the outer cell layer.

Video S1: Sporangia pressurisation. Pressurisation simulations were carried out in two steps: During cell pressurisation the cells were pressurised to 0.5MPa, whilst the inner lumen was left unpressurised, and during the lumen pressurisation stage, the lumen pressure increased by 0.01MPa. Colour represents von Mises stress measured in MPa. Scale bar = 100µm.

Video S2: Pressurisation of *Tortilicaulis-like 1*. Note the torquing motion during the cell pressurisation stage. This counteracts stress accumulation when the sporangium unravels during lumen pressurisation. Colour represents von Mises stress measured in MPa. Scale bar = 100µm.

Species	Volume of Sporangium Cavity (μm^3)	Sporangium Sphericity	Sporangium Flatness	Cell Volume (μm^3)	Cell Volume CV	Cell Anisotropy	Angle of Cell Twist (Degrees)
Cooksonioid 1	26,380,000	0.84	2.27	4,117.63	0.84	1.75	3.57
Cooksonioid 2	21,191,900	0.70	4.34	3,739.28	0.87	1.81	1.25
Ficoiditheca aenigma	19,432,000	0.89	0.88	11,385.19	0.60	2.77	1.40
Fusiform sporangium 4	5,464,940	0.72	0.38	13,652.48	0.61	2.72	6.86
Partitatheca cymosa	16,235,900	0.69	0.21	21,101.54	0.60	3.65	0.23
Fusiform sporangium 1	27,079,700	0.72	0.26	12,762.09	0.62	2.70	0.65
Fusiform sporangium 2	22,016,000	0.81	0.43	16,146.61	0.58	2.45	0.09
Fusiform sporangium 3	23,415,800	0.80	0.41	16,731.14	0.58	3.03	3.05
Partitatheca splendida	25,504,200	0.77	0.39	14,604.85	0.59	2.16	0.15
Spherical sporangium 1	10,509,500	0.88	0.71	7,971.80	0.64	2.21	0.21
Spherical sporangium 2	12,180,100	0.91	1.06	5,863.69	0.68	2.18	1.71
Spherical sporangium 3	11,304,200	0.89	0.85	6,344.16	0.67	2.29	5.37
Tortilicaulis-like 1	13,387,500	0.83	0.33	5,929.78	0.55	3.54	34.98
Tortilicaulis-like 2	16,403,900	0.85	0.40	5,866.85	0.68	3.57	31.39
Tortilicaulis-like 3	15,888,100	0.87	0.49	4,994.74	0.59	3.59	40.07

Species	CV at 0.2MPa	CV at 0.3MPa	CV at 0.4MPa	Cell Pressurisation Slope	Cell Pressurisation R^2	Sporangium Cavity Pressurisation Slope	Sporangium Cavity Pressurisation R^2	Complete Pressurisation Linear Slope	Complete Pressurisation R^2
Cooksonioid 1	0.35	0.37	0.38	-0.11	0.01	0.24	0.05	0.12	0.13
Cooksonioid 2	0.47	0.49	0.51	-0.15	0.03	0.18	0.03	-0.01	0.00
Ficoiditheca aenigma	0.32	0.35	0.35	0.24	0.02	-0.29	0.02	-0.06	0.01
Fusiform sporangium 4	0.34	0.37	0.39	-9.19	0.51	6.22	0.03	-3.03	0.01
Partitatheca cymosa	0.36	0.42	0.46	-0.75	0.01	-1.18	0.00	-1.92	0.00
Fusiform sporangium 1	0.40	0.45	0.48	-0.16	0.00	-0.31	0.00	-0.47	0.00
Fusiform sporangium 2	0.31	0.34	0.36	-0.09	0.00	0.13	0.00	0.03	0.00
Fusiform sporangium 3	0.30	0.36	0.38	-2.49	0.28	2.14	0.02	-0.38	0.00
Partitatheca splendida	0.32	0.36	0.39	2.47	0.15	-2.91	0.01	-0.43	0.00
Spherical sporangium 1	0.27	0.28	0.29	-0.85	0.03	0.98	0.02	0.11	0.00
Spherical sporangium 2	0.25	0.26	0.26	0.28	0.01	-0.37	0.01	-0.07	0.00
Spherical sporangium 3	0.27	0.29	0.29	-1.55	0.22	1.64	0.04	0.06	0.00
Tortilicaulis-like 1	0.36	0.40	0.42	-24.62	0.96	29.64	0.84	4.85	0.09
Tortilicaulis-like 2	0.36	0.41	0.43	-21.54	0.97	24.68	0.91	2.98	0.07
Tortilicaulis-like 3	0.37	0.40	0.41	-16.55	0.97	21.02	0.69	4.40	0.08

Species	Rupture Pressure (MPa)	Volume of Sporangium Cavity at Rupture (μm^3)	Stress Assisted Rupture (MPa)	Normalised Rupture Pressure	Normalised Volume of Sporangium Cavity at Rupture	Normalised Stress Assisted Rupture	Fitness
Cooksonioid 1	0.24	52,635,400	7.78	0.29	0.73	1.00	0.73
Cooksonioid 2	0.17	66,961,000	7.01	0.00	1.00	0.94	0.79
Ficoiditheca aenigma	0.28	30,761,800	5.99	0.46	0.31	0.86	0.59
Fusiform sporangium 4	0.31	14,313,600	-2.52	0.58	0.00	0.22	0.36
Partitatheca cymosa	0.26	36,693,400	-3.77	0.38	0.43	0.13	0.34
Fusiform sporangium 1	0.24	59,701,200	-1.53	0.29	0.86	0.30	0.55
Fusiform sporangium 2	0.27	42,343,000	-5.43	0.42	0.53	0.00	0.39
Fusiform sporangium 3	0.28	48,572,900	-5.26	0.46	0.65	0.01	0.46
Partitatheca splendida	0.23	54,702,500	-3.85	0.25	0.77	0.12	0.47
Spherical sporangium 1	0.41	18,540,500	-1.67	1.00	0.08	0.28	0.60
Spherical sporangium 2	0.40	20,384,300	3.36	0.96	0.12	0.67	0.68
Spherical sporangium 3	0.39	19,374,100	2.16	0.92	0.10	0.57	0.63
Tortilicaulis-like 1	0.37	22,220,000	-4.28	0.83	0.15	0.09	0.49
Tortilicaulis-like 2	0.35	27,066,900	-5.00	0.75	0.24	0.03	0.46
Tortilicaulis-like 3	0.34	26,168,300	-3.67	0.71	0.23	0.13	0.44

Table S1: Summary of statistics and measurements used in this study. The top table contains morphological and cell data used in Figure 1 and Supplementary Figure 2. The middle table shows the coefficient of variation (CV = standard deviation / mean) of the stress distributions produced in the pressurisation experiment, shown in Figure 2, and the linear regression information of the angular displacement in each sporangium, shown in Figure 3. The bottom table shows the estimated rupture pressure (also shown in Figure 2), lumen at rupture and stress-assisted rupture (also shown in Figure 4) used to calculate fitness. Those values were normalised, and fitness was calculated using Euclidian distance (see Material and Methods).

References:

Algeo, T.J. and Scheckler, S.E. 1998. Terrestrial-marine teleconnections in the Devonian: links between the evolution of land plants, weathering processes, and marine anoxic events. *Philosophical Transactions of the Royal Society of London. Series B: Biological Sciences* 353(1365), pp. 113–130. Available at: https://doi.org/10.1098/rstb.1998.0195.

Bai, Q. and Bai, Y. 2014. 9 - Thermal Expansion Design. In: Bai, Q., Bai Analysis, and Installation, Y. B. T.-S. P. D. eds. Boston: Gulf Professional Publishing, pp. 187–220. Available at: https://www.sciencedirect.com/science/article/pii/B9780123868886000092.

Bassel, G.W. et al. 2014. Mechanical constraints imposed by 3D cellular geometry and arrangement modulate growth patterns in the emArabidopsis/em embryo. *Proceedings of the National Academy of Sciences* 111(23), pp. 8685 LP – 8690. Available at: http://www.pnas.org/content/111/23/8685.abstract.

Bateman, R.M., Crane, P.R., DiMichele, W.A., Kenrick, P.R., Rowe, N.P., Speck, T. and Stein, W.E. 1998. Early evolution of land plants: phylogeny, physiology, and ecology of the primary terrestrial radiation. *Annual Review of Ecology and Systematics* 29(1), pp. 263–292.

Beauzamy, L., Louveaux, M., Hamant, O. and Boudaoud, A. 2015. Mechanically, the Shoot Apical Meristem of Arabidopsis Behaves like a Shell Inflated by a Pressure of About 1 MPa . *Frontiers in Plant Science* 6, p. 1038. Available at: https://www.frontiersin.org/article/10.3389/fpls.2015.01038.

Beerling, D.J., Osborne, C.P. and Chaloner, W.G. 2001. Evolution of leaf-form in land plants linked to atmospheric CO2 decline in the Late Palaeozoic era. *Nature* 410(6826), pp. 352–354.

Boyce, C.K. 2008. How green was Cooksonia? The importance of size in understanding the early evolution of physiology in the vascular plant lineage. *Paleobiology* 34(2), pp. 179–194. Available at: https://doi.org/10.1666/0094-8373(2008)034[0179:HGWCTI]2.0.CO.

Burns, S.J. 2015. The Theory of Materials Failure, by Richard M. Christensen. *Contemporary Physics* 56(3), p. 404. Available at: https://doi.org/10.1080/00107514.2015.1049209.

Cichan, M.A. 1986. Conductance in the wood of selected Carboniferous plants. *Paleobiology* 12(3), pp. 302–310.

Clark, J.W. et al. 2022. The origin and evolution of stomata. *Current Biology* 32(11), pp. R539–R553. Available at: https://doi.org/10.1016/j.cub.2022.04.040.

Davies, N.S., Berry, C.M., Marshall, J.E.A., Wellman, C.H. and Lindemann, F.-J. 2021. The Devonian landscape factory: plant–sediment interactions in the Old Red Sandstone of Svalbard and the rise of vegetation as a biogeomorphic agent. *Journal of the Geological Society* 178(5).

Edwards, D. 1996. New insights into early land ecosystems: a glimpse of a lilliputian world. *Review of Palaeobotany and Palynology* 90(3), pp. 159–174. Available at: https://www.sciencedirect.com/science/article/pii/003466679500081X.

Edwards, D. 2014. Cryptospores and cryptophytes reveal hidden diversity in early land floras. *New Phytologist* 202(January), pp. 50–78. doi: 10.1111/nph.12645.

Edwards, D., Fanning, U. and Richardson, J.B. 1994. Lower Devonian coalified sporangia from Shropshire: Salopella Edwards & Richardson and Tortilicaulis Edwards. *Botanical Journal of the Linnean Society* 116(2), pp. 89–110. Available at: https://doi.org/10.1111/j.1095-8339.1994.tb00425.x.

Edwards, D. and Feehan, J. 1980. Records of Cooksonia-type sporangia from late Wenlock strata in Ireland. *Nature* 287(5777), pp. 41–42. Available at: https://doi.org/10.1038/287041a0.

Edwards, D., Kenrick, P. and Dolan, L. 2017. History and contemporary significance of the Rhynie cherts—our earliest preserved terrestrial ecosystem. *Philosophical Transactions of the Royal Society B: Biological Sciences* 373(1739), p. 20160489. Available at: https://doi.org/10.1098/rstb.2016.0489.
Edwards, D., Morris, J.L., Axe, L. and Duckett, J.G. 2022a. Picking up the pieces: New charcoalified plant mesofossils (eophytes) from a Lower Devonian Lagerstätte in the Welsh Borderland, UK. *Review of Palaeobotany and Palynology* 297, p. 104567. Available at: https://www.sciencedirect.com/science/article/pii/S0034666721001913.
Edwards, D., Morris, J.L., Axe, L., Duckett, J.G., Pressel, S. and Kenrick, P. 2022b. Piecing together the eophytes – a new group of ancient plants containing cryptospores. *New Phytologist* 233(3), pp. 1440–1455. Available at: https://doi.org/10.1111/nph.17703.
Edwards, D., Morris, J.L., Axe, L., Taylor, W.A., Duckett, J.G., Kenrick, P. and Pressel, S. 2022c. Earliest record of transfer cells in Lower Devonian plants. *New Phytologist* 233(3), pp. 1456–1465. Available at: https://doi.org/10.1111/nph.17704.
Edwards, D., Richardson, J.B., Axe, L. and Davies, K.L. 2012. A new group of Early Devonian plants with valvate sporangia containing sculptured permanent dyads. *Botanical Journal of the Linnean Society*, pp. 229–257. Available at: https://doi.org/10.1111/j.1095-8339.2011.01207.x.
Edwards, D. and Wellman, C. 2001. 2. Embryophytes on Land: The Ordovician to Lochkovian (Lower Devonian) Record. In: *Plants invade the land*. Columbia University Press, pp. 3–28.
Edwards, D.S. 1986. Aglaophyton major, a non-vascular land-plant from the Devonian Rhynie Chert. *Botanical Journal of the Linnean Society* 93(2), pp. 173–204. Available at: https://doi.org/10.1111/j.1095-8339.1986.tb01020.x.
Fanning, U., Edwards, D. and Richardson, J.B. 1992. A diverse assemblage of early land plants from the Lower Devonian of the Welsh Borderland. *Botanical Journal of the Linnean Society* 109(2), pp. 161–188.
Farquhar, T. and Zhao, Y. 2006. Fracture Mechanics and Its Relevance to Botanical Structures. *American Journal of Botany* 93(10), pp. 1449–1454. Available at: http://www.jstor.org/stable/4123128.
Gess, R.W. and Prestianni, C. 2021. An early Devonian flora from the Baviaanskloof Formation (Table Mountain Group) of South Africa. *Scientific Reports* 11(1), p. 11859. Available at: https://doi.org/10.1038/s41598-021-90180-z.
Gibling, M.R. and Davies, N.S. 2012. Palaeozoic landscapes shaped by plant evolution. *Nature Geoscience* 5(2), pp. 99–105. Available at: https://doi.org/10.1038/ngeo1376.
Glasspool, I.J., Edwards, D. and Axe, L. 2004. Charcoal in the Silurian as evidence for the earliest wildfire. *Geology* 32(5), pp. 381–383. Available at: https://doi.org/10.1130/G20363.1.
Gregory, P.Herries. 1973. *The microbiology of the atmosphere*. London: L. Hill.
Habgood, K.S., Edwards, D. and Axe, L. 2002. New perspectives on Cooksonia from the Lower Devonian of the Welsh Borderland. *Botanical Journal of the Linnean Society* 139(4), pp. 339–359. Available at: https://doi.org/10.1046/j.1095-8339.2002.00073.x.
Harris, B.J., Harrison, C.J., Hetherington, A.M. and Williams, T.A. 2020. Phylogenomic Evidence for the Monophyly of Bryophytes and the Reductive Evolution of Stomata. *Current Biology* 30(11), pp. 2001–2012.e2. Available at: https://doi.org/10.1016/j.cub.2020.03.048.
Hervieux, N. et al. 2016. A Mechanical Feedback Restricts Sepal Growth and Shape in Arabidopsis. *Current Biology* 26(8), pp. 1019–1028. Available at: http://dx.doi.org/10.1016/j.cub.2016.03.004.

Hetherington, A.J. and Dolan, L. 2019. Rhynie chert fossils demonstrate the independent origin and gradual evolution of lycophyte roots. *Current Opinion in Plant Biology* 47, pp. 119–126. Available at: https://www.sciencedirect.com/science/article/pii/S1369526618300992.

Hofhuis, H. et al. 2016. Morphomechanical Innovation Drives Explosive Seed Dispersal. *Cell* 166(1), pp. 222–233. Available at: https://doi.org/10.1016/j.cell.2016.05.002.

Kaufman, L. and Rousseeuw, P.J. 2009. *Finding groups in data: an introduction to cluster analysis*. John Wiley & Sons.

Kenrick, P., Wellman, C.H., Schneider, H. and Edgecombe, G.D. 2012. A timeline for terrestrialization: consequences for the carbon cycle in the Palaeozoic. *Philosophical Transactions of the Royal Society B: Biological Sciences* 367(1588), pp. 519–536. Available at: https://doi.org/10.1098/rstb.2011.0271.

Kerp, H. 2017. Organs and tissues of Rhynie chert plants. *Philosophical Transactions of the Royal Society B: Biological Sciences* 373(1739), p. 20160495. Available at: https://doi.org/10.1098/rstb.2016.0495.

Kidston, R. and Lang, W.H. 1921. XXXII.—On Old Red Sandstone Plants showing Structure, from the Rhynie Chert Bed, Aberdeenshire. Part IV. Restorations of the Vascular Cryptogams, and Discussion of their bearing on the General Morphology of the Pteridophyta and the Origin of the Organisation of Land-Plants. *Earth and Environmental Science Transactions of The Royal Society of Edinburgh* 52(4), pp. 831–854. Available at: https://www.cambridge.org/core/article/xxxiion-old-red-sandstone-plants-showing-structure-from-the-rhynie-chert-bed-aberdeenshire-part-iv-restorations-of-the-vascular-cryptogams-and-discussion-of-their-bearing-on-the-general-morphology-of-the-pteridophyta-and-the-origin-of-the-organisation-of-landplants/B49DF14DD6C5A6BB1B7F39263B9A4ECD.

Kotyk, M.E., Basinger, J.F., Gensel, P.G. and de Freitas, T.A. 2002. Morphologically complex plant macrofossils from the Late Silurian of Arctic Canada. *American Journal of Botany* 89(6), pp. 1004–1013. Available at: https://doi.org/10.3732/ajb.89.6.1004.

Lande, R., Engen, S. and Sæther, B.-E. 2009. An evolutionary maximum principle for density-dependent population dynamics in a fluctuating environment. *Philosophical Transactions of the Royal Society B: Biological Sciences* 364(1523), pp. 1511–1518. Available at: https://doi.org/10.1098/rstb.2009.0017.

Lenton, T.M., Dahl, T.W., Daines, S.J., Mills, B.J.W., Ozaki, K., Saltzman, M.R. and Porada, P. 2016. Earliest land plants created modern levels of atmospheric oxygen. *Proceedings of the National Academy of Sciences* 113(35), pp. 9704–9709. Available at: https://doi.org/10.1073/pnas.1604787113.

Li, C.-S. and Edwards, D. 1992. A new genus of early land plants with novel strobilar construction from the Lower Devonian Posongchong Formation, Yunnan Province, China. *Palaeontology* 35(2), pp. 257–272.

Libertín, M., Kvaček, J., Bek, J., Žárský, V. and Štorch, P. 2018. Sporophytes of polysporangiate land plants from the early Silurian period may have been photosynthetically autonomous. *Nature Plants* 4(5), pp. 269–271. Available at: https://doi.org/10.1038/s41477-018-0140-y.

Lloyd, S. 1982. Least squares quantization in PCM. *IEEE transactions on information theory* 28(2), pp. 129–137.

Long, Y. et al. 2020. Cellular Heterogeneity in Pressure and Growth Emerges from Tissue Topology and Geometry. *Current Biology* 30(8), pp. 1504-1516.e8. Available at: https://www.sciencedirect.com/science/article/pii/S0960982220302001.

Majda, M., Trozzi, N., Mosca, G. and Smith, R.S. 2022. How Cell Geometry and Cellular Patterning Influence Tissue Stiffness. *International Journal of Molecular Sciences* 23(10). doi: 10.3390/ijms23105651.

McDaniel, S.F. 2021. Bryophytes are not early diverging land plants. *New Phytologist* 230(4), pp. 1300–1304. Available at: https://doi.org/10.1111/nph.17241.

McMahon, W.J. and Davies, N.S. 2018. Evolution of alluvial mudrock forced by early land plants. *Science* 359(6379), pp. 1022–1024. Available at: https://doi.org/10.1126/science.aan4660.

Morris, J.L. et al. 2015. Investigating Devonian trees as geo-engineers of past climates: linking palaeosols to palaeobotany and experimental geobiology. *Palaeontology* 58(5), pp. 787–801. Available at: https://doi.org/10.1111/pala.12185.

Morris, J.L., Edwards, D., Richardson, J.B. and Axe, L. 2012. New dyad-producing plants from the Lower Devonian (Lochkovian) of the Welsh Borderland. *Botanical Journal of the Linnean Society* 169(4), pp. 569–595. Available at: https://doi.org/10.1111/j.1095-8339.2012.01231.x.

Mosca, G., Sapala, A., Strauss, S., Routier-Kierzkowska, A.-L. and Smith, R.S. 2017. On the micro-indentation of plant cells in a tissue context. *Physical Biology* 14(1), p. 15003. Available at: http://dx.doi.org/10.1088/1478-3975/aa5698.

Nawaschin, S. 1897. Ueber die Sporenausschleuderung bei den Torfmoosen. *Flora* 83, pp. 151–159.

Niklas, K.J. 1992. *Plant biomechanics: an engineering approach to plant form and function*. University of Chicago press.

Noblin, X., Rojas, N.O., Westbrook, J., Llorens, C., Argentina, M. and Dumais, J. 2012. The Fern Sporangium: A Unique Catapult. *Science* 335(6074), p. 1322. Available at: https://doi.org/10.1126/science.1215985.

Pan, R.Z. and Dong, Y.D. 1994. Plant Physiology, vol. 2.

Pirani, J.R. and Prado, J. 2012. Embryopsida, a new name for the class of land plants. *Taxon* 61(5), pp. 1096–1098.

Puttick, M.N. et al. 2018. The Interrelationships of Land Plants and the Nature of the Ancestral Embryophyte. *Current Biology* 28(5), pp. 733–745.e2. Available at: https://doi.org/10.1016/j.cub.2018.01.063.

Remy, W. 1982. Lower Devonian Gametophytes: Relation to the Phylogeny of Land Plants. *Science* 215(4540), pp. 1625–1627. Available at: https://doi.org/10.1126/science.215.4540.1625.

Richardson, J.. B. and McGregor, D.C. 1986. *Silurian and Devonian spore zones of the Old Red Sandstone Continent and adjacent regions*. Ottawa, Canada: Geological Survey of Canada.

Robinson, H. and Shaw, J. 1984. Considerations on the Evolution of the Moss Operculum. *The Bryologist* 87(4), pp. 293–296. Available at: http://www.jstor.org/stable/3242946.

Roper, M., Seminara, A., Bandi, M.M., Cobb, A., Dillard, H.R. and Pringle, A. 2010. Dispersal of fungal spores on a cooperatively generated wind. *Proceedings of the National Academy of Sciences* 107(41), pp. 17474–17479. Available at: https://doi.org/10.1073/pnas.1003577107.

Sapala, A. et al. 2018. Why plants make puzzle cells, and how their shape emerges. McCormick, S. ed. *eLife* 7, p. e32794. Available at: https://doi.org/10.7554/eLife.32794.

Schneller, J., Gerber, H. and Zuppiger, A. 2008. Speed and force of spore ejection in Selaginella martensii. *Botanica Helvetica* 118(1), pp. 13–20. Available at: https://doi.org/10.1007/s00035-008-0814-6.

Shah, D.U. 2013. Developing plant fibre composites for structural applications by optimising composite parameters: a critical review. *Journal of Materials Science* 48(18), pp. 6083–6107. Available at: https://doi.org/10.1007/s10853-013-7458-7.

Shou-Gang, H. and Gensel, P.G. 2001. 6. The Posongchong Floral Assemblages of Southeastern Yunnan, China—Diversity and Disparity in Early Devonian Plant Assemblages. In: Gensel, P. G. and Edwards, D. eds. *Evolutionary and Environmental Perspectives*. Columbia University Press, pp. 103–119. Available at: https://doi.org/10.7312/gens11160-007.

Sokal, R. 1958. A statiscal method for evaluating systematic relationships. *Univ Kans sci bull* 38, pp. 1409–1438.

de Sousa, F., Foster, P.G., Donoghue, P.C.J., Schneider, H. and Cox, C.J. 2019. Nuclear protein phylogenies support the monophyly of the three bryophyte groups (Bryophyta Schimp.). *New Phytologist* 222(1), pp. 565–575. Available at: https://doi.org/10.1111/nph.15587.

Tarnita, C.E., Washburne, A., Martinez-Garcia, R., Sgro, A.E. and Levin, S.A. 2015. Fitness tradeoffs between spores and nonaggregating cells can explain the coexistence of diverse genotypes in cellular slime molds. *Proceedings of the National Academy of Sciences* 112(9), pp. 2776–2781. Available at: https://doi.org/10.1073/pnas.1424242112.

Taylor, T.N., Kerp, H. and Hass, H. 2005. Life history biology of early land plants: Deciphering the gametophyte phase. *Proceedings of the National Academy of Sciences* 102(16), pp. 5892–5897. Available at: https://doi.org/10.1073/pnas.0501985102.

Taylor, T.N., Klavins, S.D., Krings, M., Taylor, E.L., Kerp, H. and Hass, H. 2003. Fungi from the Rhynie chert: a view from the dark side. *Earth and Environmental Science Transactions of The Royal Society of Edinburgh* 94(4), pp. 457–473. Available at: https://www.cambridge.org/core/article/fungi-from-the-rhynie-chert-a-view-from-the-dark-side/92F8EAE63680C25D55420362275ADEA1.

Tomescu, A.M.F. 2022. Mysteries of the bryophyte–tracheophyte transition revealed: enter the eophytes. *New Phytologist* 233(3), pp. 1018–1021. Available at: https://doi.org/10.1111/nph.17876.

Tomescu, A.M.F., Wyatt, S.E., Hasebe, M. and Rothwell, G.W. 2014. Early evolution of the vascular plant body plan — the missing mechanisms. *Current Opinion in Plant Biology* 17, pp. 126–136. Available at: https://www.sciencedirect.com/science/article/pii/S1369526613001933.

Wallace, M.W., Hood, A. vS., Shuster, A., Greig, A., Planavsky, N.J. and Reed, C.P. 2017. Oxygenation history of the Neoproterozoic to early Phanerozoic and the rise of land plants. *Earth and Planetary Science Letters* 466, pp. 12–19. Available at: https://www.sciencedirect.com/science/article/pii/S0012821X1730122X.

Weber, A., Braybrook, S., Huflejt, M., Mosca, G., Routier-Kierzkowska, A.-L. and Smith, R.S. 2015. Measuring the mechanical properties of plant cells by combining micro-indentation with osmotic treatments. *Journal of Experimental Botany* 66(11), pp. 3229–3241. Available at: https://doi.org/10.1093/jxb/erv135.

Weiss-Lehman, C. and Shaw, A.K. 2022. Understanding the drivers of dispersal evolution in range expansions and their ecological consequences. *Evolutionary Ecology* 36(2), pp. 181–197. Available at: https://doi.org/10.1007/s10682-022-10166-9.

Wellman, C.H., Edwards, D. and Axe, L. 1998. Ultrastructure of laevigate hilate spores in sporangia and spore masses from the Upper Silurian and Lower Devonian of the Welsh Borderland. *Philosophical Transactions of the Royal Society of London. Series B: Biological Sciences* 353(1378), pp. 1983–2004.

Whitaker, D.L. and Joan, E. 2010. Sphagnum Moss Disperses Spores with Vortex Rings. *Science* 329(5990), p. 406. Available at: https://doi.org/10.1126/science.1190179.

Wilson, J.P. and Fischer, W.W. 2011. Hydraulics of Asteroxylon mackei, an early Devonian vascular plant, and the early evolution of water transport tissue in terrestrial plants. *Geobiology* 9(2), pp. 121–130.

Wilson, J.P., Knoll, A.H., Holbrook, N.M. and Marshall, C.R. 2008. Modeling fluid flow in Medullosa, an anatomically unusual Carboniferous seed plant. *Paleobiology* 34(4), pp. 472–493.

Yafetto, L. et al. 2008. The Fastest Flights in Nature: High-Speed Spore Discharge Mechanisms among Fungi. *PLOS ONE* 3(9), p. e3237. Available at: https://doi.org/10.1371/journal.pone.0003237.

Zhang, Y., Yu, J., Wang, X., Durachko, D.M., Zhang, S. and Cosgrove, D.J. 2021. Molecular insights into the complex mechanics of plant epidermal cell walls. *Science* 372(6543), pp. 706–711. Available at: https://doi.org/10.1126/science.abf2824.

Algeo, T.J. and Scheckler, S.E. 1998. Terrestrial-marine teleconnections in the Devonian: links between the evolution of land plants, weathering processes, and marine anoxic events. *Philosophical Transactions of the Royal Society of London. Series B: Biological Sciences* 353(1365), pp. 113–130. Available at: https://doi.org/10.1098/rstb.1998.0195.

Bai, Q. and Bai, Y. 2014. 9 - Thermal Expansion Design. In: Bai, Q., Bai Analysis, and Installation, Y. B. T.-S. P. D. eds. Boston: Gulf Professional Publishing, pp. 187–220. Available at: https://www.sciencedirect.com/science/article/pii/B9780123868886000092.

Bassel, G.W. et al. 2014. Mechanical constraints imposed by 3D cellular geometry and arrangement modulate growth patterns in the emArabidopsis/em embryo. *Proceedings of the National Academy of Sciences* 111(23), pp. 8685 LP – 8690. Available at: http://www.pnas.org/content/111/23/8685.abstract.

Bateman, R.M., Crane, P.R., DiMichele, W.A., Kenrick, P.R., Rowe, N.P., Speck, T. and Stein, W.E. 1998. Early evolution of land plants: phylogeny, physiology, and ecology of the primary terrestrial radiation. *Annual Review of Ecology and Systematics* 29(1), pp. 263–292.

Beauzamy, L., Louveaux, M., Hamant, O. and Boudaoud, A. 2015. Mechanically, the Shoot Apical Meristem of Arabidopsis Behaves like a Shell Inflated by a Pressure of About 1 MPa . *Frontiers in Plant Science* 6, p. 1038. Available at: https://www.frontiersin.org/article/10.3389/fpls.2015.01038.

Beerling, D.J., Osborne, C.P. and Chaloner, W.G. 2001. Evolution of leaf-form in land plants linked to atmospheric CO2 decline in the Late Palaeozoic era. *Nature* 410(6826), pp. 352–354.

Boyce, C.K. 2008. How green was Cooksonia? The importance of size in understanding the early evolution of physiology in the vascular plant lineage. *Paleobiology* 34(2), pp. 179–194. Available at: https://doi.org/10.1666/0094-8373(2008)034[0179:HGWCTI]2.0.CO.

Burns, S.J. 2015. The Theory of Materials Failure, by Richard M. Christensen. *Contemporary Physics* 56(3), p. 404. Available at: https://doi.org/10.1080/00107514.2015.1049209.

Cichan, M.A. 1986. Conductance in the wood of selected Carboniferous plants. *Paleobiology* 12(3), pp. 302–310.

Clark, J.W. et al. 2022. The origin and evolution of stomata. *Current Biology* 32(11), pp. R539–R553. Available at: https://doi.org/10.1016/j.cub.2022.04.040.

Davies, N.S., Berry, C.M., Marshall, J.E.A., Wellman, C.H. and Lindemann, F.-J. 2021. The Devonian landscape factory: plant–sediment interactions in the Old Red Sandstone of Svalbard and the rise of vegetation as a biogeomorphic agent. *Journal of the Geological Society* 178(5).

Edwards, D. 1996. New insights into early land ecosystems: a glimpse of a lilliputian world. *Review of Palaeobotany and Palynology* 90(3), pp. 159–174. Available at: https://www.sciencedirect.com/science/article/pii/003466679500081X.

Edwards, D. 2014. Cryptospores and cryptophytes reveal hidden diversity in early land floras. *New Phytologist* 202(January), pp. 50–78. doi: 10.1111/nph.12645.

Edwards, D., Fanning, U. and Richardson, J.B. 1994. Lower Devonian coalified sporangia from Shropshire: Salopella Edwards & Richardson and Tortilicaulis Edwards. *Botanical Journal of the Linnean Society* 116(2), pp. 89–110. Available at: https://doi.org/10.1111/j.1095-8339.1994.tb00425.x.

Edwards, D. and Feehan, J. 1980. Records of Cooksonia-type sporangia from late Wenlock strata in Ireland. *Nature* 287(5777), pp. 41–42. Available at: https://doi.org/10.1038/287041a0.

Edwards, D., Kenrick, P. and Dolan, L. 2017. History and contemporary significance of the Rhynie cherts—our earliest preserved terrestrial ecosystem. *Philosophical Transactions of the Royal Society B: Biological Sciences* 373(1739), p. 20160489. Available at: https://doi.org/10.1098/rstb.2016.0489.

Edwards, D., Morris, J.L., Axe, L. and Duckett, J.G. 2022a. Picking up the pieces: New charcoalified plant mesofossils (eophytes) from a Lower Devonian Lagerstätte in the Welsh Borderland, UK. *Review of Palaeobotany and Palynology* 297, p. 104567. Available at: https://www.sciencedirect.com/science/article/pii/S0034666721001913.

Edwards, D., Morris, J.L., Axe, L., Duckett, J.G., Pressel, S. and Kenrick, P. 2022b. Piecing together the eophytes – a new group of ancient plants containing cryptospores. *New Phytologist* 233(3), pp. 1440–1455. Available at: https://doi.org/10.1111/nph.17703.

Edwards, D., Morris, J.L., Axe, L., Taylor, W.A., Duckett, J.G., Kenrick, P. and Pressel, S. 2022c. Earliest record of transfer cells in Lower Devonian plants. *New Phytologist* 233(3), pp. 1456–1465. Available at: https://doi.org/10.1111/nph.17704.

Edwards, D., Richardson, J.B., Axe, L. and Davies, K.L. 2012. A new group of Early Devonian plants with valvate sporangia containing sculptured permanent dyads. *Botanical Journal of the Linnean Society*, pp. 229–257. Available at: https://doi.org/10.1111/j.1095-8339.2011.01207.x.

Edwards, D. and Wellman, C. 2001. 2. Embryophytes on Land: The Ordovician to Lochkovian (Lower Devonian) Record. In: *Plants invade the land*. Columbia University Press, pp. 3–28.

Edwards, D.S. 1986. Aglaophyton major, a non-vascular land-plant from the Devonian Rhynie Chert. *Botanical Journal of the Linnean Society* 93(2), pp. 173–204. Available at: https://doi.org/10.1111/j.1095-8339.1986.tb01020.x.

Fanning, U., Edwards, D. and Richardson, J.B. 1992. A diverse assemblage of early land plants from the Lower Devonian of the Welsh Borderland. *Botanical Journal of the Linnean Society* 109(2), pp. 161–188.

Farquhar, T. and Zhao, Y. 2006. Fracture Mechanics and Its Relevance to Botanical Structures. *American Journal of Botany* 93(10), pp. 1449–1454. Available at: http://www.jstor.org/stable/4123128.

Gess, R.W. and Prestianni, C. 2021. An early Devonian flora from the Baviaanskloof Formation (Table Mountain Group) of South Africa. *Scientific Reports* 11(1), p. 11859. Available at: https://doi.org/10.1038/s41598-021-90180-z.

Gibling, M.R. and Davies, N.S. 2012. Palaeozoic landscapes shaped by plant evolution. *Nature Geoscience* 5(2), pp. 99–105. Available at: https://doi.org/10.1038/ngeo1376.

Glasspool, I.J., Edwards, D. and Axe, L. 2004. Charcoal in the Silurian as evidence for the earliest wildfire. *Geology* 32(5), pp. 381–383. Available at: https://doi.org/10.1130/G20363.1.

Gregory, P.Herries. 1973. *The microbiology of the atmosphere*. London: L. Hill.

Habgood, K.S., Edwards, D. and Axe, L. 2002. New perspectives on Cooksonia from the Lower Devonian of the Welsh Borderland. *Botanical Journal of the Linnean Society* 139(4), pp. 339–359. Available at: https://doi.org/10.1046/j.1095-8339.2002.00073.x.

Harris, B.J., Harrison, C.J., Hetherington, A.M. and Williams, T.A. 2020. Phylogenomic Evidence for the Monophyly of Bryophytes and the Reductive Evolution of Stomata. *Current Biology* 30(11), pp. 2001–2012.e2. Available at: https://doi.org/10.1016/j.cub.2020.03.048.

Hervieux, N. et al. 2016. A Mechanical Feedback Restricts Sepal Growth and Shape in Arabidopsis. *Current Biology* 26(8), pp. 1019–1028. Available at: http://dx.doi.org/10.1016/j.cub.2016.03.004.

Hetherington, A.J. and Dolan, L. 2019. Rhynie chert fossils demonstrate the independent origin and gradual evolution of lycophyte roots. *Current Opinion in Plant Biology* 47, pp. 119–126. Available at: https://www.sciencedirect.com/science/article/pii/S1369526618300992.

Hofhuis, H. et al. 2016. Morphomechanical Innovation Drives Explosive Seed Dispersal. *Cell* 166(1), pp. 222–233. Available at: https://doi.org/10.1016/j.cell.2016.05.002.

Kaufman, L. and Rousseeuw, P.J. 2009. *Finding groups in data: an introduction to cluster analysis*. John Wiley & Sons.

Kenrick, P., Wellman, C.H., Schneider, H. and Edgecombe, G.D. 2012. A timeline for terrestrialization: consequences for the carbon cycle in the Palaeozoic. *Philosophical Transactions of the Royal Society B: Biological Sciences* 367(1588), pp. 519–536. Available at: https://doi.org/10.1098/rstb.2011.0271.

Kerp, H. 2017. Organs and tissues of Rhynie chert plants. *Philosophical Transactions of the Royal Society B: Biological Sciences* 373(1739), p. 20160495. Available at: https://doi.org/10.1098/rstb.2016.0495.

Kidston, R. and Lang, W.H. 1921. XXXII.—On Old Red Sandstone Plants showing Structure, from the Rhynie Chert Bed, Aberdeenshire. Part IV. Restorations of the Vascular Cryptogams, and Discussion of their bearing on the General Morphology of the Pteridophyta and the Origin of the Organisation of Land-Plants. *Earth and Environmental Science Transactions of The Royal Society of Edinburgh* 52(4), pp. 831–854. Available at: https://www.cambridge.org/core/article/xxxiion-old-red-sandstone-plants-showing-structure-from-the-rhynie-chert-bed-aberdeenshire-part-iv-restorations-of-the-vascular-cryptogams-and-discussion-of-their-bearing-on-the-general-morphology-of-the-pteridophyta-and-the-origin-of-the-organisation-of-landplants/B49DF14DD6C5A6BB1B7F39263B9A4ECD.

Kotyk, M.E., Basinger, J.F., Gensel, P.G. and de Freitas, T.A. 2002. Morphologically complex plant macrofossils from the Late Silurian of Arctic Canada. *American Journal of Botany* 89(6), pp. 1004–1013. Available at: https://doi.org/10.3732/ajb.89.6.1004.

Lande, R., Engen, S. and Sæther, B.-E. 2009. An evolutionary maximum principle for density-dependent population dynamics in a fluctuating environment. *Philosophical Transactions of the Royal Society B: Biological Sciences* 364(1523), pp. 1511–1518. Available at: https://doi.org/10.1098/rstb.2009.0017.

Lenton, T.M., Dahl, T.W., Daines, S.J., Mills, B.J.W., Ozaki, K., Saltzman, M.R. and Porada, P. 2016. Earliest land plants created modern levels of atmospheric oxygen. *Proceedings of the National Academy of Sciences* 113(35), pp. 9704–9709. Available at: https://doi.org/10.1073/pnas.1604787113.

Li, C.-S. and Edwards, D. 1992. A new genus of early land plants with novel strobilar construction from the Lower Devonian Posongchong Formation, Yunnan Province, China. *Palaeontology* 35(2), pp. 257–272.

Libertín, M., Kvaček, J., Bek, J., Žárský, V. and Štorch, P. 2018. Sporophytes of polysporangiate land plants from the early Silurian period may have been photosynthetically autonomous. *Nature Plants* 4(5), pp. 269–271. Available at: https://doi.org/10.1038/s41477-018-0140-y.

Lloyd, S. 1982. Least squares quantization in PCM. *IEEE transactions on information theory* 28(2), pp. 129–137.

Long, Y. et al. 2020. Cellular Heterogeneity in Pressure and Growth Emerges from Tissue Topology and Geometry. *Current Biology* 30(8), pp. 1504-1516.e8. Available at: https://www.sciencedirect.com/science/article/pii/S0960982220302001.

Majda, M., Trozzi, N., Mosca, G. and Smith, R.S. 2022. How Cell Geometry and Cellular Patterning Influence Tissue Stiffness. *International Journal of Molecular Sciences* 23(10). doi: 10.3390/ijms23105651.

McDaniel, S.F. 2021. Bryophytes are not early diverging land plants. *New Phytologist* 230(4), pp. 1300–1304. Available at: https://doi.org/10.1111/nph.17241.

McMahon, W.J. and Davies, N.S. 2018. Evolution of alluvial mudrock forced by early land plants. *Science* 359(6379), pp. 1022–1024. Available at: https://doi.org/10.1126/science.aan4660.

Morris, J.L. et al. 2015. Investigating Devonian trees as geo-engineers of past climates: linking palaeosols to palaeobotany and experimental geobiology. *Palaeontology* 58(5), pp. 787–801. Available at: https://doi.org/10.1111/pala.12185.

Morris, J.L., Edwards, D., Richardson, J.B. and Axe, L. 2012. New dyad-producing plants from the Lower Devonian (Lochkovian) of the Welsh Borderland. *Botanical Journal of the Linnean Society* 169(4), pp. 569–595. Available at: https://doi.org/10.1111/j.1095-8339.2012.01231.x.

Mosca, G., Sapala, A., Strauss, S., Routier-Kierzkowska, A.-L. and Smith, R.S. 2017. On the micro-indentation of plant cells in a tissue context. *Physical Biology* 14(1), p. 15003. Available at: http://dx.doi.org/10.1088/1478-3975/aa5698.

Nawaschin, S. 1897. Ueber die Sporenausschleuderung bei den Torfmoosen. *Flora* 83, pp. 151–159.

Niklas, K.J. 1992. *Plant biomechanics: an engineering approach to plant form and function*. University of Chicago press.

Noblin, X., Rojas, N.O., Westbrook, J., Llorens, C., Argentina, M. and Dumais, J. 2012. The Fern Sporangium: A Unique Catapult. *Science* 335(6074), p. 1322. Available at: https://doi.org/10.1126/science.1215985.

Pan, R.Z. and Dong, Y.D. 1994. Plant Physiology, vol. 2.

Pirani, J.R. and Prado, J. 2012. Embryopsida, a new name for the class of land plants. *Taxon* 61(5), pp. 1096–1098.

Puttick, M.N. et al. 2018. The Interrelationships of Land Plants and the Nature of the Ancestral Embryophyte. *Current Biology* 28(5), pp. 733–745.e2. Available at: https://doi.org/10.1016/j.cub.2018.01.063.

Remy, W. 1982. Lower Devonian Gametophytes: Relation to the Phylogeny of Land Plants. *Science* 215(4540), pp. 1625–1627. Available at: https://doi.org/10.1126/science.215.4540.1625.

Richardson, J.. B. and McGregor, D.C. 1986. *Silurian and Devonian spore zones of the Old Red Sandstone Continent and adjacent regions*. Ottawa, Canada: Geological Survey of Canada.

Robinson, H. and Shaw, J. 1984. Considerations on the Evolution of the Moss Operculum. *The Bryologist* 87(4), pp. 293–296. Available at: http://www.jstor.org/stable/3242946.

Roper, M., Seminara, A., Bandi, M.M., Cobb, A., Dillard, H.R. and Pringle, A. 2010. Dispersal of fungal spores on a cooperatively generated wind. *Proceedings of the National Academy of Sciences* 107(41), pp. 17474–17479. Available at: https://doi.org/10.1073/pnas.1003577107.

Sapala, A. et al. 2018. Why plants make puzzle cells, and how their shape emerges. McCormick, S. ed. *eLife* 7, p. e32794. Available at: https://doi.org/10.7554/eLife.32794.

Schneller, J., Gerber, H. and Zuppiger, A. 2008. Speed and force of spore ejection in Selaginella martensii. *Botanica Helvetica* 118(1), pp. 13–20. Available at: https://doi.org/10.1007/s00035-008-0814-6.

Shah, D.U. 2013. Developing plant fibre composites for structural applications by optimising composite parameters: a critical review. *Journal of Materials Science* 48(18), pp. 6083–6107. Available at: https://doi.org/10.1007/s10853-013-7458-7.

Shou-Gang, H. and Gensel, P.G. 2001. 6. The Posongchong Floral Assemblages of Southeastern Yunnan, China—Diversity and Disparity in Early Devonian Plant Assemblages. In: Gensel, P. G. and Edwards, D. eds. *Evolutionary and Environmental Perspectives*. Columbia University Press, pp. 103–119. Available at: https://doi.org/10.7312/gens11160-007.

Sokal, R. 1958. A statiscal method for evaluating systematic relationships. *Univ Kans sci bull* 38, pp. 1409–1438.

de Sousa, F., Foster, P.G., Donoghue, P.C.J., Schneider, H. and Cox, C.J. 2019. Nuclear protein phylogenies support the monophyly of the three bryophyte groups (Bryophyta Schimp.). *New Phytologist* 222(1), pp. 565–575. Available at: https://doi.org/10.1111/nph.15587.

Tarnita, C.E., Washburne, A., Martinez-Garcia, R., Sgro, A.E. and Levin, S.A. 2015. Fitness tradeoffs between spores and nonaggregating cells can explain the coexistence of diverse genotypes in cellular slime molds. *Proceedings of the National Academy of Sciences* 112(9), pp. 2776–2781. Available at: https://doi.org/10.1073/pnas.1424242112.

Taylor, T.N., Kerp, H. and Hass, H. 2005. Life history biology of early land plants: Deciphering the gametophyte phase. *Proceedings of the National Academy of Sciences* 102(16), pp. 5892–5897. Available at: https://doi.org/10.1073/pnas.0501985102.

Taylor, T.N., Klavins, S.D., Krings, M., Taylor, E.L., Kerp, H. and Hass, H. 2003. Fungi from the Rhynie chert: a view from the dark side. *Earth and Environmental Science Transactions of The Royal Society of Edinburgh* 94(4), pp. 457–473. Available at: https://www.cambridge.org/core/article/fungi-from-the-rhynie-chert-a-view-from-the-dark-side/92F8EAE63680C25D55420362275ADEA1.

Tomescu, A.M.F. 2022. Mysteries of the bryophyte–tracheophyte transition revealed: enter the eophytes. *New Phytologist* 233(3), pp. 1018–1021. Available at: https://doi.org/10.1111/nph.17876.

Tomescu, A.M.F., Wyatt, S.E., Hasebe, M. and Rothwell, G.W. 2014. Early evolution of the vascular plant body plan — the missing mechanisms. *Current Opinion in Plant Biology* 17, pp. 126–136. Available at: https://www.sciencedirect.com/science/article/pii/S1369526613001933.

Wallace, M.W., Hood, A. vS., Shuster, A., Greig, A., Planavsky, N.J. and Reed, C.P. 2017. Oxygenation history of the Neoproterozoic to early Phanerozoic and the rise of land plants. *Earth and Planetary Science Letters* 466, pp. 12–19. Available at: https://www.sciencedirect.com/science/article/pii/S0012821X1730122X.

Weber, A., Braybrook, S., Huflejt, M., Mosca, G., Routier-Kierzkowska, A.-L. and Smith, R.S. 2015. Measuring the mechanical properties of plant cells by combining micro-indentation with osmotic treatments. *Journal of Experimental Botany* 66(11), pp. 3229–3241. Available at: https://doi.org/10.1093/jxb/erv135.

Weiss-Lehman, C. and Shaw, A.K. 2022. Understanding the drivers of dispersal evolution in range expansions and their ecological consequences. *Evolutionary Ecology* 36(2), pp. 181–197. Available at: https://doi.org/10.1007/s10682-022-10166-9.

Wellman, C.H., Edwards, D. and Axe, L. 1998. Ultrastructure of laevigate hilate spores in sporangia and spore masses from the Upper Silurian and Lower Devonian of the Welsh Borderland. *Philosophical Transactions of the Royal Society of London. Series B: Biological Sciences* 353(1378), pp. 1983–2004.

Whitaker, D.L. and Joan, E. 2010. Sphagnum Moss Disperses Spores with Vortex Rings. *Science* 329(5990), p. 406. Available at: https://doi.org/10.1126/science.1190179.

Wilson, J.P. and Fischer, W.W. 2011. Hydraulics of Asteroxylon mackei, an early Devonian vascular plant, and the early evolution of water transport tissue in terrestrial plants. *Geobiology* 9(2), pp. 121–130.

Wilson, J.P., Knoll, A.H., Holbrook, N.M. and Marshall, C.R. 2008. Modeling fluid flow in Medullosa, an anatomically unusual Carboniferous seed plant. *Paleobiology* 34(4), pp. 472–493.

Yafetto, L. et al. 2008. The Fastest Flights in Nature: High-Speed Spore Discharge Mechanisms among Fungi. *PLOS ONE* 3(9), p. e3237. Available at: https://doi.org/10.1371/journal.pone.0003237.

Zhang, Y., Yu, J., Wang, X., Durachko, D.M., Zhang, S. and Cosgrove, D.J. 2021. Molecular insights into the complex mechanics of plant epidermal cell walls. *Science* 372(6543), pp. 706–711. Available at: https://doi.org/10.1126/science.abf2824.
